# Powdered Cross-Linked Gelatin Methacryloyl as an Injectable Hydrogel for Adipose Tissue Engineering

**DOI:** 10.3390/gels10030167

**Published:** 2024-02-26

**Authors:** Tess De Maeseneer, Lana Van Damme, Merve Kübra Aktan, Annabel Braem, Paula Moldenaers, Sandra Van Vlierberghe, Ruth Cardinaels

**Affiliations:** 1 Soft Matter, Rheology and Technology, Department of Chemical Engineering, KU Leuven, Celestijnenlaan 200J Box 2424, 3001 Leuven, Belgium; tess.demaeseneer@kuleuven.be (T.D.M.); paula.moldenaers@kuleuven.be (P.M.); 2 Polymer Chemistry & Biomaterials Group, Centre of Macromolecular Chemistry, Department of Organic and Macromolecular Chemistry, Ghent University (UGent), Krijgslaan 281, S4-Bis, 9000 Ghent, Belgium; lana.vandamme@ugent.be (L.V.D.); sandra.vanvlierberghe@ugent.be (S.V.V.); 3 Biomaterials and Tissue Engineering Research Group, Department of Materials Engineering, KU Leuven, Kasteelpark Arenberg 44 Box 2450, 3001 Leuven, Belgium; mervekubra.aktan@kuleuven.be (M.K.A.); annabel.braem@kuleuven.be (A.B.); 4 Processing and Performance of Materials, Department of Mechanical Engineering, TU Eindhoven, P.O. Box 513, 5600 MB Eindhoven, The Netherlands

**Keywords:** adipose tissue, tissue engineering, cross-linked gelatin methacryloyl, particulate hydrogel, injectable hydrogel

## Abstract

The tissue engineering field is currently advancing towards minimally invasive procedures to reconstruct soft tissue defects. In this regard, injectable hydrogels are viewed as excellent scaffold candidates to support and promote the growth of encapsulated cells. Cross-linked gelatin methacryloyl (GelMA) gels have received substantial attention due to their extracellular matrix-mimicking properties. In particular, GelMA microgels were recently identified as interesting scaffold materials since the pores in between the microgel particles allow good cell movement and nutrient diffusion. The current work reports on a novel microgel preparation procedure in which a bulk GelMA hydrogel is ground into powder particles. These particles can be easily transformed into a microgel by swelling them in a suitable solvent. The rheological properties of the microgel are independent of the particle size and remain stable at body temperature, with only a minor reversible reduction in elastic modulus correlated to the unfolding of physical cross-links at elevated temperatures. Salts reduce the elastic modulus of the microgel network due to a deswelling of the particles, in addition to triple helix denaturation. The microgels are suited for clinical use, as proven by their excellent cytocompatibility. The latter is confirmed by the superior proliferation of encapsulated adipose tissue-derived stem cells in the microgel compared to the bulk hydrogel. Moreover, microgels made from the smallest particles are easily injected through a 20G needle, allowing a minimally invasive delivery. Hence, the current work reveals that powdered cross-linked GelMA is an excellent candidate to serve as an injectable hydrogel for adipose tissue engineering.

## 1. Introduction

Currently, there exists a great need for tissue engineering (TE) strategies to reconstruct soft tissue damage and defects that accompany congenital defects, disease or trauma [1]. The reconstructive methods that are currently widely applied, such as implants and autologous fat grafting, suffer from serious drawbacks, including infection, scar formation, necrosis and pain [2]. Therefore, the reconstructive field is evolving towards minimally invasive procedures, with adipose tissue engineering taking part in this trend. Adipose tissue engineering combines adipose tissue-derived stem cells (ASCs) and a scaffold material to create newly grown soft tissue [3]. The scaffold aims at supporting and nurturing the cells inside the body so that they can proliferate and differentiate into tissue, after which the scaffold degrades [4]. To deliver the scaffold to the site of interest, the most promising strategy involves the use of injectable materials [5,6]. Due to their flowability combined with having a gel character at rest, these materials are able to adapt to very complex patient-specific voids. Moreover, they avoid the need for open surgery, which greatly contributes to their minimally invasive character [5]. To design a suitable injectable material for adipose tissue engineering, a well-defined set of requirements needs to be fulfilled. In addition to excellent injectability for straightforward administration, the scaffold needs to interact with the surrounding tissue and promote the growth of the encapsulated cells while it resides in the physiological environment that is defined as the extracellular matrix (ECM). It is therefore imperative that the scaffold is biocompatible and maintains its stability by exhibiting adequate mechanical properties in physiological conditions for an appropriate period of time [7].

Throughout the past decades, polymeric hydrogels have emerged as promising scaffold candidates, mainly due to their ECM-mimicking properties [6,8]. Natural hydrogels are generally preferred due to their high tendency towards biocompatibility, with prevalent examples being chitosan, alginate, hyaluronic acid, collagen and gelatin [9]. Within this list, collagen and gelatin, the latter being a derivative of collagen, are deemed the most interesting since collagen is one of the main constituents of the ECM [1,10]. Furthermore, the backbone of both polymers contains the Arg-Gly-Asp (RGD) amino acid triplet, which promotes cell adhesion [11]. On the other hand, natural polymers are known to be characterized by poor mechanical properties. However, this problem can be easily tackled via enzymatic cross-linking or through chemical modifications [12,13]. Van Den Bulcke et al. [14] were the first to propose the chemical modification of gelatin with methacrylic anhydride to obtain cross-linkable gelatin methacryloyl. The induced chemical cross-links provide additional strength and make sure that the gelatin maintains its gel character at temperatures above its physical melting temperature of about 30 °C. Many authors have already reported on the successful use of cross-linked gelatin methacryloyl (GelMA) gels in tissue engineering, biofabrication and drug delivery [15,16,17,18].

In addition to the use of bulk GelMA hydrogels in biomedical applications, the use of GelMA-based microgels has also been explored by multiple researchers [19,20,21,22]. Microgels can be defined as suspensions of soft polymeric gel particles swollen in a solvent. If these particles reach a closely packed state or are highly attractive, the suspension obtains an elastic character, thereby transforming the viscous-like suspension into an elastic gel or paste [23]. The final elastic properties of the gel highly depend on the total volume occupied by the particles as well as their mechanical properties. It is, however, very difficult to determine the particle volume fraction as well as how close it is to the maximum packing since microgel particles are able to deform. This enables them to occupy much larger volume fractions than particles in hard sphere suspensions, which have a maximum packing fraction of about 0.64 for a random packing [24]. Due to this deformation, packing fractions ranging from 0.64 to 1 can be obtained. These high packing fractions cause a very steep increase in the elastic modulus of the particle network [25]. Packing fractions can even extend beyond unity due to the deswelling of the particles. Polyelectrolyte particles in particular show this phenomenon, whereby osmotic deswelling is induced via the diffusion of free counter-ions into the solvent [25]. In addition to volume fraction, the individual particle mechanics also determine the stiffness of a microgel, with stiffer particles resulting in stiffer microgels. These particle mechanics are dictated by the cross-linking density of the polymers [26]. Adams et al. [27] have compared the elastic moduli of microgels made from particles with different stiffnesses, showing that stiffer particles with higher polymer concentrations, and hence more cross-links, can result in a gel elastic modulus of up to two orders of magnitude higher than soft particles for the same volume fraction of particles. Furthermore, the deswelling of the particles also alters the individual particle mechanics, with collapsed particles typically being stiffer than their swollen counterparts [28]. This means that the packing-induced deswelling of the particles at the maximum packing fraction will cause an even further increase in the gel’s elastic modulus [25,29]. Even though the use of GelMA microgels has been widely reported, no in-depth rheological investigations have been performed on this microgel. Specifically, most authors only report the bulk shear modulus of the microgel without correlating this to volume fraction, swelling or individual particle mechanics.

The use of microgels instead of bulk hydrogels is extremely interesting due to the high adaptability of their mechanical properties, which can be easily achieved by altering their volume fraction or particle modulus. In addition, microgels possess many biological advantages over bulk hydrogels, especially for tissue engineering applications [30]. The main issue related to cell encapsulation inside a bulk hydrogel is the confined cell movement and limited nutrient diffusion through the small pores [31]. The interstitial space between microgel particles enhances its porosity, thereby positively impacting cell growth [30]. Even though the use of GelMA microgels has been explored previously, these microgels were mostly fabricated by similar methods, more specifically microfluidics, emulsification, electrostatic droplet generation, two-step desolvation or complex coacervation [19,20,21,22,32,33,34,35]. These techniques allow precise control of the particle properties, but the throughput is typically low. Table 1 summarizes the most commonly applied methods and the main drawbacks associated with them. Mechanical fragmentation in the wet state was applied by a few authors to other hydrogel types. To the best of our knowledge, none of the works report on the use of powdered cross-linked gelatin methacryloyl microgels created by grinding a bulk GelMA hydrogel. However, through this technique, high-throughput and low-cost production of GelMA microgels is possible. Therefore, the current work aims to evaluate the potential of powdered cross-linked gelatin methacryloyl as an injectable hydrogel for adipose tissue engineering. The discussion starts with an in-depth description of the powder particle preparation procedure, followed by a thorough characterization of the obtained product. Next, the gels made by dissolving the created powder particles in a suitable solvent are evaluated in terms of rheological stability at physiological conditions, represented by elevated temperature and ionic strength, injectability and cytocompatibility.

## 2. Results and Discussion

### 2.1. Development and Characterization of the GelMA Precursors

#### 2.1.1. Degree of Substitution (DS)

The DS of the obtained GelMA was determined via ^1^H-NMR spectroscopy, as shown in Figure S1 in the Supplementary Information, by assessing the characteristic MA peaks at 5.6 and 5.8 ppm compared to the peak of the hydrogens corresponding to the chemically inert methyl groups of the amino acids valine, leucine and isoleucine at 1.01 ppm. A DS of 63% (0.24 mmol methacryloyl/g gelatin) was successfully obtained, which is in accordance with previous research [39].

#### 2.1.2. Molar Mass (MM)

GPC measurements were performed to assess the influence of the functionalization of the gelatin backbone in terms of molar mass (MM). The results indicate a slight decrease (13%) in MM (from 162,100 ± 18,200 Da to 141,300 ± 16,800 Da) following synthesis, which can potentially be attributed to hydrolysis. It can be noted that Van Hoorick et al. [40] described a decrease of 7.5% in MM under similar reaction conditions. The polydispersity of GelB was 2.39 ± 0.09 compared to 2.51 ± 0.06 for the modified gelatin.

#### 2.1.3. Melting Temperature and Enthalpy

**Figure 1:**
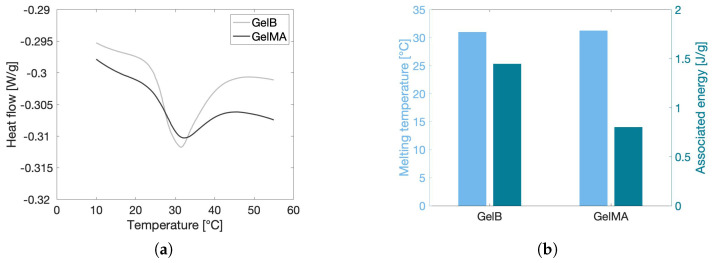
DSC−thermograms (second heating run) of both pristine GelB and GelMA in water at a concentration of 10 *w*/*v*%. (**a**) The heat flow of the second heating run is illustrated as a function of the temperature. The peak of the curve represents the melting temperature of each solution. (**b**) Both melting temperature (left axis and left bars) and associated energy (right axis and right bars) based on the second heating run are reported.

Gelatin exhibits an upper critical solution temperature (UCST), meaning that below this temperature, the biopolymer forms collagen-like triple helices, resulting in a physical network [41]. The molar mass and incorporation of moieties onto the backbone, amongst others, can negatively impact the triple helix formation and thus the UCST. Therefore, the sol–gel behavior of gelatin before and after modification was studied via DSC, which measures the difference in heat that is required to increase the temperature of the examined material and that of a reference sample. The results of this investigation are shown in Figure 1. The endothermal transitions of GelB and GelMA occurred at similar melting temperatures (∼30 °C). This implies that the triple helix formation still occurs following modification. Nonetheless, a large decrease (44.5%) in melting enthalpy could be observed. These results are in line with the work of Rebers et al. [42], in which they report an enthalpy decrease of 33.3% upon modification of gelatin. The formation of hydrogen bonds is most likely hampered due to the incorporation of the MA moieties onto the backbone, causing steric hindrance [14,42,43]. Although the molar mass can influence the melting enthalpy [44], only a limited decrease in molar mass (∼13%) could be observed according to GPC measurements (vide supra), thus limiting the contribution of this factor.

### 2.2. Characterization of the GelMA Powder Particles and the Resulting Microgel

The GelMA precursor material was chemically cross-linked and processed into a swellable powder, as described in Section 4.4. The obtained powder particles have a rectangular shape, as visualized in Figures S2–S6 in the Supplementary Information. Sieves with mesh sizes of 100 µm, 212 µm, 300 µm, 400 µm and 500 µm were used to separate the particles into five size fractions, further referred to as <100 µm, 100–212 µm, 212–300 µm, 300–400 µm and 400–500 µm.

#### 2.2.1. Particle Size Distribution (PSD)

**Figure 2:**
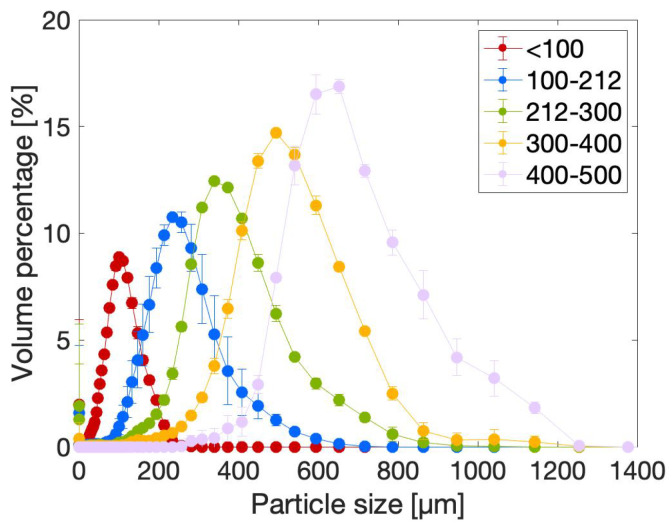
Particle size distribution curves of the dry powder particles of the size fractions <100 µm, 100–212 µm, 212–300 µm, 300–400 µm and 400–500 µm.

The particle size distributions of the five size fractions were measured by means of laser diffraction using butyldiglycol acetate as a solvent. The latter is a hydrophobic solvent that does not penetrate the hydrophilic particles, which allows for the measurement of the size of the unswollen powder particles. The results are presented in Figure 2. Each curve shows a monomodal Gaussian distribution. This indicates that sieving allows for a proper size fractionation of the particles.

#### 2.2.2. Particle Aspect Ratio

Figures S2–S6 in the Supplementary Information show microscopy images of typical particles for each size fraction. Although the particles all have a similar rectangular shape, the aspect ratio of the particles decreases with an increasing particle size. A quantification of the aspect ratios, defined as the ratio of the width and the height, can be found in Table 2. Figure S6e shows the almost square particles of a swollen 10 *w*/*v*% microgel of 400–500 µm particles.

#### 2.2.3. Effect of Particle Size on the Rheological Gel Properties

**Figure 3:**
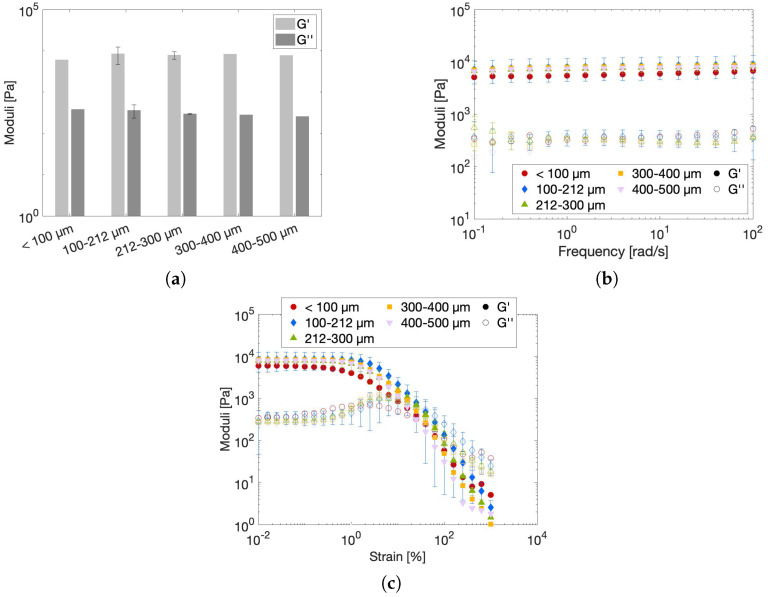
(**a**) Steady−state moduli at 0.1% strain and 10 rad/s, (**b**) frequency sweep and (**c**) strain sweep of 10 *w*/*v*% gels made from multiple size fractions of powder in water measured at 20 °C. Error bars on the hydrogels made from particles retained between sieves of 100 and 212 µm and 212 and 300 µm represent reference standard deviations.

Rheological measurements were carried out on gel samples made from particles of different sizes belonging to five size fractions, previously stated to be <100 µm, 100–212 µm, 212–300 µm, 300–400 µm and 400–500 µm. To firstly rule out the possible effects of temperature and salt concentration, it was decided to perform these measurements isothermally at 20 °C with water as the solvent. The gels were prepared inside a syringe two hours prior to the measurement to achieve complete swelling of the particles, and samples were subsequently injected onto the rheometer bottom plate. The moduli of the gels were probed for one hour to allow full restructuring of any structural breakdown that could have possibly occurred during sample loading. The steady-state moduli are presented in Figure 3a for all five size fractions. The hydrogels made from 100–212 µm and 212–300 µm powder particles were measured twice to provide error intervals for referencing. These two size fractions were chosen since the in-depth structural analysis (see Section 2.3.2 and Section 2.4) was performed on these size fractions as well. The presented moduli show no significant differences, which allows the conclusion that particle size has no effect on the steady-state stiffness of the created hydrogels. A microgel’s stiffness is determined by its occupied volume fraction relative to the maximum packing fraction and the individual particle modulus. Both are highly influenced by the degree of swelling of the particles, where a high swelling leads to more deformable particles. A highly deformable particle has a low elastic modulus, reducing the microgel’s stiffness but allowing high volume fractions to be reached, which increases the elastic modulus of the microgel. Moreover, highly swollen particles have the ability to deswell upon particle contact, which allows even higher volume fractions to be reached [25,29]. In addition to swelling, which dictates the ultimate particle size, particle size distribution (PSD) and particle shape also have an effect on the microgel’s stiffness, as both PSD and particle shape determine the maximum attainable packing fraction [45]. High packing fractions can be reached with particles that have a broad PSD without the need for particle deswelling. The PSDs of the different swollen particle size fractions are very different (Figure S7 in the Supplementary Information), as are their aspect ratios (Table 2). This means that, for each size fraction, there will be a unique yet complex interplay between the occupied volume, the individual particle modulus and the particle shape. Nonetheless, the combination of all parameters leads to the formation of gels with an elastic modulus that is insensitive to particle size and has a value around 8 kPa. This value is in between the values of 10 kPa reported by Zoratto et al. [20] for a jammed 20 wt% GelMA microgel and 2 kPa reported by [35] for a GelMA microgel bioink.

The frequency sweep, shown in Figure 3b, renders valuable information on the structure of the gel. In addition to the elastic modulus exceeding the loss modulus over the entire frequency range, the frequency independence of the elastic modulus is maintained at low frequencies, which is indicative of a true gel character [46]. Finally, the strain sweeps, presented in Figure 3c, are studied to define the linear viscoelastic (LVE) region and the yielding mechanism of the particulate gel. The LVE region can be defined as the region wherein both the elastic and loss moduli are independent of applied strain. This region extends until approximately 0.4% strain for all investigated hydrogels since, at this strain, one of the two moduli differs by more than 5% from its original linear value. Increasing the strain beyond this LVE limit leads to an overshoot in G” followed by a decrease in both G’ and G”. Although this type of yielding can be assigned to the predefined yielding class III of Hyun et al. [47], often reported for soft glassy materials including microgel particles dispersed in water, the microstructural mechanism governing this behavior is highly material-dependent [47,48,49]. Hence, no complete microstructural picture can be sketched without additional extensive non-linear investigations, which are beyond the scope of the current work.

### 2.3. Stability of the Viscoelastic Gel at Physiological Conditions

One of the main prerequisites for a material to be used as scaffold material in adipose tissue engineering applications is that it needs to maintain a stable gel elasticity under physiological conditions. The latter are mainly defined by the elevated body temperature and the salt concentrations present in body fluids at neutral to slightly alkaline pH levels [50]. To characterize the gel’s behavior under these conditions, rheological measurements were performed at temperatures covering the gel’s preparation phase at room temperature and the injection phase into the body at 37 °C, in addition to scanning the rheological properties of hydrogels made from solvents with differing salt concentrations at room temperature.

#### 2.3.1. Effect of Temperature on the Rheological Gel Properties

**Figure 4:**
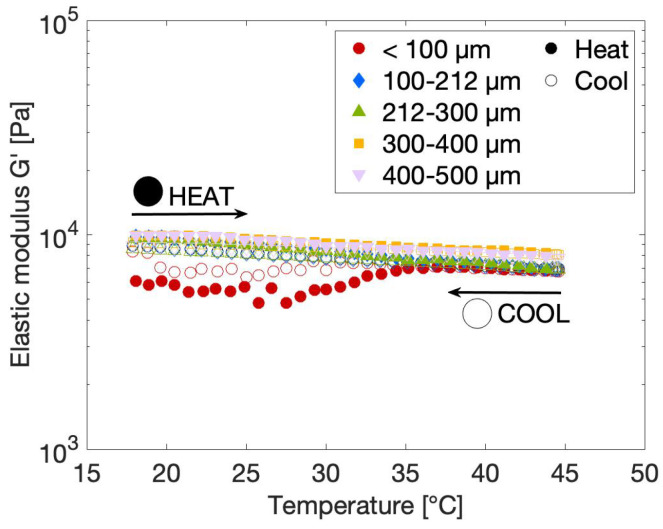
Elastic modulus G’ at a strain of 0.1% and frequency of 10 rad/s as a function of increasing (•) and decreasing (∘) temperature at 0.85 °C/min for 10 *w*/*v*% gels made from powder particles of different sizes in water.

Body temperature was previously stated to be one of the most important environmental conditions to which the scaffold material will be exposed. Therefore, the rheological properties of the gels of variable particle sizes were measured as a function of increasing and decreasing temperature, as shown in Figure 4. The temperature was varied between 18 °C and 45 °C to make sure that all temperatures encountered during tissue engineering applications, including preparation at room temperature and injection at body temperature, were covered. The graph shows that there is a small dependency of the gel structure on temperature. Upon increasing the temperature, the elastic modulus of the gels slightly decreases, with the decrease starting at a temperature of approximately 30 °C. This temperature is known to be the melting temperature of gelatin, as confirmed by the DSC results presented in Figure 1, whereby physical cross-links present as helix structures unfold into random coil chains [51]. This helix–coil transition is completely reversible, as confirmed by the rheological data that indicate that the elastic modulus is restored upon cooling the gel again to room temperature. The magnitude of the physical loss of structure is correlated to the level of chemical modification of the gel, since the substituted gelatin methacryloyl groups induce steric hindrance, thereby complicating the formation of physical cross-links [14,42]. The GelMA used in the current work has a DS of 63%, such that a substantial amount of steric hindrance imposed by the methacryloyl groups is expected. The latter is also confirmed by the DSC results, which indicate that the melting enthalpy of the gelatin substantially decreases after chemical modification, thereby confirming that fewer physical cross-links are present in the GelMA sample. To promote at least some formation of physical cross-links, the modified precursor solutions are left to rest in the fridge for one hour prior to chemical cross-linking. Nonetheless, subsequent exposure of the gels to UV light could produce the heat that might melt some of these physical cross-links. As a result, not that many triple helices are present at low temperatures, and are subsequently lost at temperatures above the sol–gel transition, resulting in a stable gel structure, as aimed for. Based on Figure 4, it can again be concluded that particle size has no to possibly a very small effect on the rheological behavior of the gels. The loss in elastic modulus seems to be slightly more pronounced for the smaller particle sizes. However, the maximum measured difference in the elastic moduli between particle sizes is 1.3 kPa, which is well below the previously defined reference measurement error of 1.5 kPa, as indicated in Figure 3a. It should be noted that the data of the <100 µm sample were very prone to scattering in the low-temperature region for unknown reasons, so no conclusions could be drawn from this.

**Figure 5:**
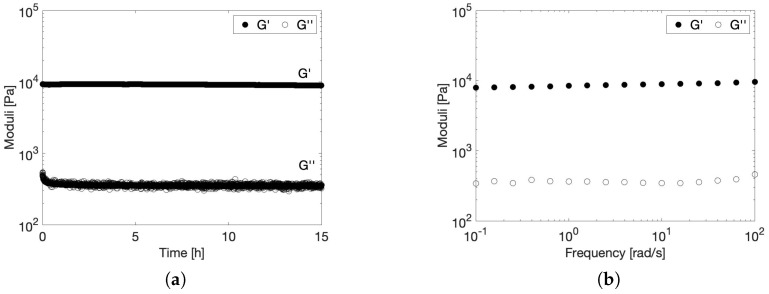
Shear−moduli of a 10 *w*/*v*% microgel constituting 212–300 µm particles in water at 37 °C. (**a**) Time sweep at 0.1% strain and 10 rad/s and (**b**) frequency sweep at 0.1% strain.

To further investigate the viscoelastic stability of the microgel when exposed to body temperature for an extended period of time, a time sweep and a frequency sweep at 37 °C were performed and are presented in Figure 5. This measurement was performed on the 212–300 µm microgel, which is representative for all other size fractions since particle size has no effect on the temperature dependency of the rheological properties, as proven in Figure 4. The time sweep in Figure 5a indicates that the gel remains stable over the entire measurement time frame of 15 h. In addition, Figure 5b indicates that the gel shows no relaxations in the measured frequency range, since both G’ and G” are independent of frequency over the entire measurement range. This further supports the excellent viscoelastic stability of the gel at body temperature.

#### 2.3.2. Effect of Ionic Strength on the Rheological Gel Properties

**Figure 6:**
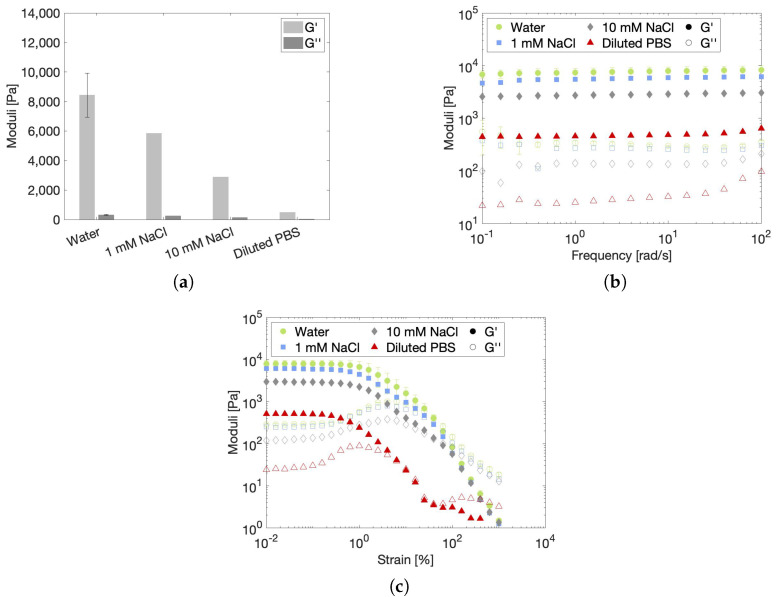
(**a**) Steady−state moduli at 0.1% strain and 10 rad/s, (**b**) frequency sweep and (**c**) strain sweep of 10 *w*/*v*% gels made from 212–300 µm powder particles in water, 1 mM NaCl, 10 mM NaCl and diluted PBS measured at 20 °C. Error bars on the hydrogel made in water represent a reference standard deviation.

A second environmental condition that needs to be investigated is the presence of salts, which alter the ionic strength of the environmental solvent in comparison to that of pure water. Upon injection inside the human body, the hydrogel scaffold will be exposed to a high concentration of salts present in body fluids, including the ECM. Ideally, the scaffold itself resembles this ECM so that encapsulated cells can reside in their natural environment, thereby facilitating their differentiation and proliferation into new tissue. This means that, from a biological point of view, a saline solution would serve better as a solvent compared to water. Therefore, the rheological properties of hydrogel samples that were prepared in different salt solutions were investigated at room temperature. Since sodium is one of the main constituents of the ECM, it was chosen to study two concentrations of sodium chlorine solution, namely 1 mM and 10 mM [52]. PBS was chosen as a third solvent because it effectively simulates in vivo conditions. The specifications of the solvents are listed in Table 3. The pH of the salt solutions was situated between 7.1 and 7.6, which perfectly matches the slightly alkaline extracellular pH of approximately 7.3–7.4 [50]. Figure 6 compares the rheological properties of hydrogels prepared in water and in the three saline solutions. Since it was previously demonstrated that the rheological properties are independent of particle size, the effect of salts was investigated on a single size fraction, arbitrarily chosen to be 212–300 µm. The steady-state moduli in Figure 6a, which are displayed according to increasing ionic strength, clearly indicate that a higher salt concentration leads to a substantial decrease in hydrogel stiffness. Nonetheless, all samples maintain the character of a true gel, as can be concluded from the frequency sweeps in Figure 6b. In addition, salts seem to have no influence on the microstructural yielding mechanism of the hydrogels since the weak G” overshoot is present in Figure 6c for all hydrogel samples. However, the LVE of the 10 mM NaCl and diluted PBS samples reduces from a 0.4% strain, measured for water and 1 mM NaCl, to a 0.2% strain. This means that the hydrogel network is more easily perturbed under strain at high salt concentrations.

### 2.4. Characterization of the Microgel Structural Properties

#### 2.4.1. Volume Fraction

**Figure 7:**
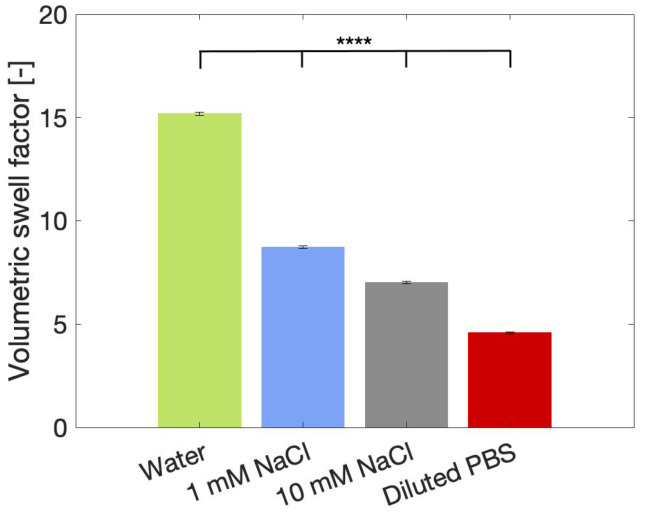
Volumetric swell factor of particles in the 212–300 µm size range recorded in water, 1 mM NaCl, 10 mM NaCl and diluted PBS. *p* < 0.0001 for all combinations indicated with ****.

To understand the mechanism underlying the hydrogel’s stiffness reduction caused by salts, all parameters that contribute to the stiffness of a particulate network should be taken into account. Previously, it was stated that the occupied volume fraction and particle packing, as well as the individual particle stiffness, determine the stiffness of the created microgel network. Both parameters were described as being highly influenced by particle swelling. Therefore, laser diffraction was performed to determine the swelling of the particles in the selected salt solutions, as presented in Figure 7. The volumetric swell factors showed a decreasing trend with increasing ionic strength, similar to the trend observed for the rheological data in Figure 6a. As all particle suspensions in Figure 7 were prepared from one single size fraction, the PSD and particle shape are the same for all solutions and should thus not be taken into account for a comparative analysis. By multiplying the dry volume of particles with the obtained swell factors, the particle volume fractions can be estimated. The dry volume is calculated based on the true density of the particles, which was measured by helium pycnometry (ρ = 1.46 g/cm^3^). Helium is unable to penetrate inside the dry particles (as concluded from BET porosity measurements), so this density value represents the particle density. The resulting volume fractions are presented in Table 4. The results show that the volume occupied by the particles decreases with increasing ionic strength, which directly translates into a less stiff network for the highly saline samples. Interestingly, the water-based hydrogel has an estimated volume fraction of 104%, indicating that some particle deswelling must occur for all the particles to fit in the volume. This packing-induced deswelling is expected to make the particles less deformable, which contributes to the elevated microgel’s stiffness.

#### 2.4.2. Individual Particle Deformability

**Figure 8:**
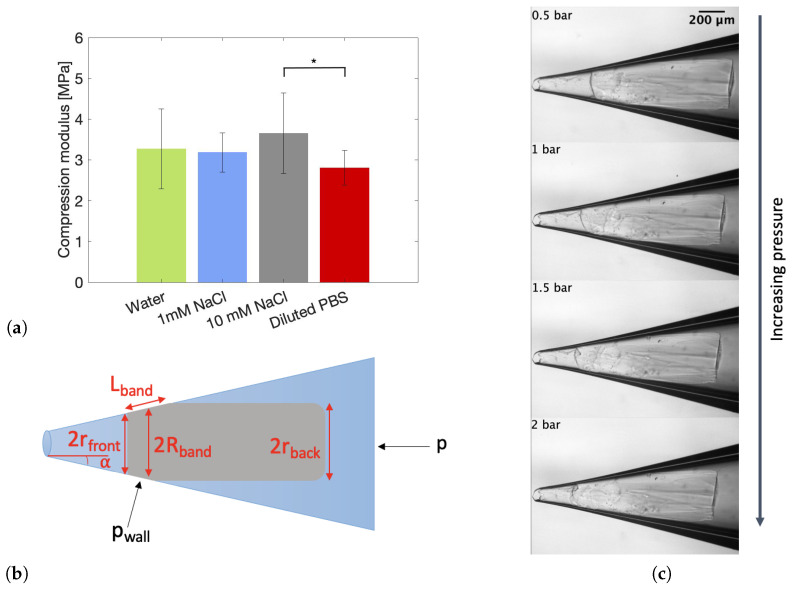
(**a**) Compression modulus of particles in the 212–300 µm size range recorded in water, 1 mM NaCl, 10 mM NaCl and diluted PBS. Statistically significant (*p* < 0.05) differences are indicated by *. (**b**) Graphical representation of a particle captured in a tapered capillary with all necessary parameters for calculations indicated. (**c**) Micromechanical images of a particle compressed at multiple pressures in a tapered capillary (in 1 mM NaCl).

The reduced swelling of the particles as a function of salt concentration might impact the individual particle deformability. To verify this hypothesis, micromechanical measurements were performed. In brief, the procedure encompasses the entrapment and subsequent compression of a swollen particle in a tapered capillary. By capturing images of the deformed particle under various pressures, the compression modulus of the particle can be calculated. In theory, the shear modulus can also be calculated from these images. However, this was impossible for the powder particles in the current study due to their complex shape (see Figures S2–S6 in the Supplementary Information). The calculated compression moduli for particles swollen in various solvents can be found in Figure 8a. All parameters necessary for the calculations are indicated in Figure 8b. The raw pressure-compression data, showing a linear relation from which the moduli can be calculated, can be found in Figure S8 in the Supplementary Information. In addition, an exemplary image series of a particle swollen in 1 mM NaCl and deformed at multiple pressures is shown in Figure 8c. Figure 8a indicates that the relative error for this measurement and/or calculation is quite large, which is attributed to the irregular shape of the particles, making it difficult to evaluate the exact volumetric change. Nonetheless, conclusions can be drawn from the data by applying the relevant statistics. No differences in compression modulus are detected for the particles swollen in water, 1 mM NaCl and 10 mM NaCl. On the other hand, it is statistically proven that the compression modulus of a particle swollen in diluted PBS is significantly lower than that of a particle swollen in 10 mM NaCl. Interestingly, this indicates that particles in a more swollen state are more able to withstand compression than particles in a collapsed or unswollen state. This result is counterintuitive since it is expected that particles in diluted PBS, which are the least swollen, are characterized by the highest compression modulus. Consequently, it is hypothesized that the gel’s material properties change due to the addition of salt. Several authors have reported on the reduction of the stiffness of regular gelatin with the addition of salt above a certain concentration [53,54,55,56].

#### 2.4.3. Helix Denaturation and Zeta Potential

**Figure 9:**
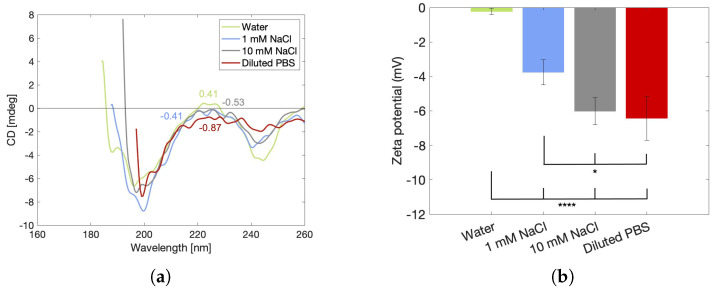
(**a**) CD spectra with the peak values at a wavelength of 222 nm indicated and (**b**) apparent zeta potential of particles in the 100–212 µm size range swollen in water, 1 mM NaCl, 10 mM NaCl and diluted PBS measured at room temperature. Statistically significant (*p* < 0.05) differences are indicated by asterisks (* = *p* < 0.05, **** = *p* < 0.0001).

Two hypotheses have been proposed to explain this stiffness reduction. The first one states that the ions impede the formation of hydrogen bonds, which inhibits the formation of triple helices [53,55]. These helices contribute to the stiffness of gelatin, as shown in the work of Rebers et al. [57] and as demonstrated here in Section 2.3.1, where it is shown that the loss of physical cross-links at elevated temperatures causes a reduction in the elastic modulus. The validity of this first hypothesis is tested here by performing circular dichroism (CD) measurements to provide information on the folding of the gelatin chains. Stable, folded structures in the form of triple helices can be recognized by a positive band at 222 nm in the CD spectrum. Denaturation of the helices into unfolded chains is characterized by a shift of this band to negative values that decrease further with more chains being unfolded [58,59]. The obtained CD spectra of 100–212 µm particles swollen in water and in all three salt solutions are presented in Figure 9a. It should be noted that here particles within the 100–212 µm range are chosen to minimize sedimentation inside the cuvette during the measurement. However, since particle size does not affect the rheological properties of the microgel, it is also not expected that there will be conformational differences between the particle sizes. Therefore, these measurements can be used to explain the rheological outcomes of the 212–300 µm particles swollen in various solutions presented in Figure 6. The water-swollen particles have a triple helix structure that can be deduced from the positive band at 222 nm. This band decreases with increasing salt content, indicating that salt ions clearly destabilize the existing helices present in the powder particles and/or inhibit the formation of new helices, which could potentially form due to the increased mobility induced by swelling of the powder particles. Therefore, the particles swollen in saline solutions with high ionic strength are slightly softer compared to those in water.

A second hypothesis to explain the salt-induced stiffness reduction in gelatin has also been reported in the literature. This hypothesis suggests that the salts bind to the available charged groups of gelatin, some of which are positively and some of which are negatively charged according to the amphoteric nature of the gelatin, thereby shielding the short-range electrostatic interactions to ultimately promote aggregation of the polymer chains [54,56]. This aggregation is linked to salting out, whereby water and the aggregates are phase separated. For a particulate gel, salting out leads to a decrease in the size of the particles [60]. This effect was also observed in the tested hydrogels (see Figure 7), supporting the validity of the hypothesis. However, to fully confirm the idea of electrostatic shielding by salts, zeta potential measurements were performed on powder particles in water, NaCl solutions and diluted PBS. The results are presented in Figure 9b. The obtained zeta potential values are negative for all samples and increase in absolute magnitude with increasing ionic strengths. At neutral pH, gelatin methacryloyl based on gelatin type B is expected to contain an overall negative charge since the isoelectric point of gelatin type B is situated around pH 5 [61]. Moreover, the binding of the methacryloyl groups on the positively charged amines of gelatin further increases the overall negative charge. The obtained value for the particles swollen in water is very close to zero, whereas literature reports values around −8 mV for gelatin methacryloyl solutions based on gelatin type B [62]. It should be noted that it was very challenging to obtain stable and reliable results for the particles in water. The instability of the raw data of particles in water compared to particles in salt solutions is visualized in the Supplementary Materials (Figure S9). Furthermore, it is counterintuitive that an increased amount of salts increases the absolute magnitude of the zeta potential since it is expected that salts would screen the remaining negative charges, thereby making the polymer chains more neutral, such that they can aggregate. This means that there must be an additional phenomenon occurring that has an impact on these measurements. It is well known that the magnitude of the zeta potential is highly impacted by the bulk swelling of the material. When the material swells, the interface between the solid surface and the electrolyte becomes less distinct, which reduces the electrokinetic effect and, as a result, the magnitude of the zeta potential [63]. This means that instead of measuring the actual zeta potential of the polymer chains, an apparent zeta potential that is inversely proportional to the volumetric swelling factor is measured. Hence, the zeta potential results should not be interpreted as a measure for the interparticle interactions but rather as a confirmation of the obtained swelling degrees of the particles. Due to this interference of the particle swelling, data on the particle charge are not available.

To conclude, the addition of salts leads to a reduced volume fraction and, at high salt contents, a softening of the particles. Both effects contribute to the reduction of the elastic modulus of the microgel network. Since salts are inevitably present inside the human body and the scaffold material should mimic the ECM, which also contains high salt concentrations, it is logical to create a hydrogel in a saline solution. Furthermore, cell proliferation and differentiation are optimal when the elastic modulus of the scaffold equals the elastic modulus of the actual tissue [64]. Values in between 0.8 and 2 kPa have been reported for adipose or breast tissue [64,65]. These values are in between the values obtained for 10 mM NaCl and diluted PBS, indicating that, also from a mechanical point of view, salt solutions prove to be the best suited solvent for adipose tissue engineering. Interestingly, the elastic modulus of a bulk GelMA hydrogel of similar DS and concentration is determined to be 14 kPa (see Figure S10 in the Supplementary Information). This means that, based on its mechanical properties, the powdered microgel is better suited as a scaffold for adipose tissue engineering than the bulk hydrogel. Moreover, salt concentration can be used as a parameter to fine-tune the mechanical properties of the scaffold. However, if this concentration is fixed to be the same as that of the ECM, it is still possible to optimize the mechanical properties by changing the particle volume fraction via the concentration of particles based on the determined volumetric swell factor. Hence, volume fraction is an additional design parameter that gives the microgel more design freedom than a bulk GelMA hydrogel. Furthermore, the saline microgels have a LVE region that spans until 0.2%, as determined from Figure 6c. This LVE limit of 0.2% strain is similar to the value of 0.1% strain that is reported for subcutaneous adipose tissue by Geerligs et al. [66] and for human abdominal adipose tissue by Patel et al. [67]. It should be noted that both of these measurements were performed at a temperature of 37 °C, whilst the measurement in Figure 6c was performed at 20 °C. However, it was confirmed, based on Figure S11 in the Supplementary Information, that a temperature change from 20 °C to 37 °C has no effect on the yielding of the microgel. Hence, it can be concluded that the microgels are able to withstand similar strains compared to actual tissue, making them suitable for temporary replacement.

### 2.5. Injectability of the Gel

#### 2.5.1. Force–Displacement Measurement

**Figure 10:**
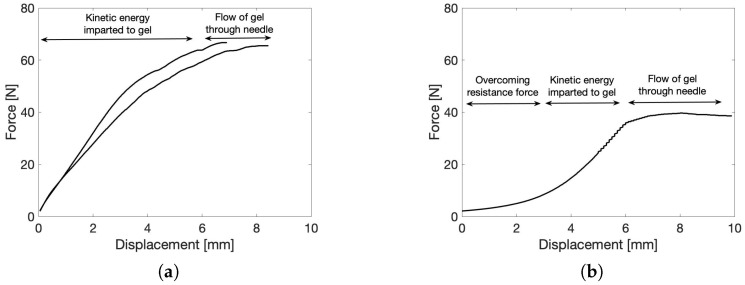
Injectability of the microgel at room temperature via mechanical testing using a 1 mL syringe equipped with a 20G (inner diameter 603 µm) needle under two conditions. (**a**) Slow injection, pushing 1 mL of 100–212 µm microgel in water in 42 s through the needle. (**b**) Fast injection, pushing 1 mL of <120 µm microgel in water in 21 s through the needle.

Since the minimally invasive delivery of a biomaterial is of utmost importance for the translational capability of the material towards the clinic, an injectability study was performed. The maximal required load force for the injection of materials in vivo can vary greatly depending on the specific material being injected and the method of delivery. In general, the maximal load force should be carefully considered and optimized to ensure that the material can be delivered safely and effectively to the in vivo environment. Here, two relevant injection conditions were defined and tested on the microgels prepared in water. The first condition encompasses a slow injection, in which 1 mL of material is injected in 42 s. For the second condition, a faster injection was tested, more specifically an injection of 1 mL of material in 21 s. To this end, a syringe plunger was compressed by the crosshead of a tensile tester at a set rate to achieve the defined conditions, and the required force was measured. It turned out that the slow injection through a 20G (inner diameter 603 µm) needle was impossible for microgels constituting particles larger than 212 µm. These larger particles could, however, be injected through a syringe without a needle, as shown in Figure S12 in the Supplementary Information. In addition, these curves indicate that the maximum force necessary to inject the microgels highly depends on the particle size, with smaller particles being more easily injected. The swell factor of the particles swollen in water equals approximately 15, which means that the size of the particles increases with this factor. The micromechanical measurements (Figure 8a) made it clear that the particles are highly incompressible when swollen in water, which means that their size will almost not change during injection. Therefore, only the dimensions of the swollen particles smaller than 212 µm are small enough to be pushed through the 603 µm needle opening. Since the particles have a smaller swell factor (Figure 7) and are more compressible (Figure 8a) in salt solutions, it is expected that larger particles can be injected through a 20G needle when swollen in a saline solution. The largest water-swollen particles that could be injected through the 20G needle are in the 100–212 µm size fraction; these results are presented in duplicate in Figure 10a. A video supporting the injectability of this gel is provided in the Supplementary Information. In addition, images are provided in the Supplementary Information in Figure S3 that indicate that injection through a needle has no effect on the particle morphology. In general, the injectability data can be divided into three regions. In the first region, the resistance force of the plunger needs to be overcome, which is followed by the second region, in which kinetic energy will be imparted to the solution. Lastly, the solution will be steadily forced through the needle [68]. Figure 10a shows that the sample readily overcomes the resistance force and reaches the last region, indicated by the plateau. The 100–212 µm sample shows an F*_max_* of 66.1 ± 0.8 N. By quantifying the maximum force needed for injection and correlating it to a subjective ease of injection (including the force correlated to the wall friction of the plunger), the potential for clinical translation could be confirmed. This can be further strengthened by the average maximum force that can be generated manually being 79.8 N on average [69]. Hence, the average human is able to inject this material into a patient with ease. Next, the fast injection condition was tested on the microgels. Now, only the microgels constituting particles <120 µm were injectable through a 20G needle. The resulting force–displacement curve is presented in Figure 10b. This curve shows that a faster injection requires a larger displacement to overcome the resistance force of the plunger. However, after overcoming this force, the curve readily transitions into the last region, in which the microgel steadily flows through the needle. Then, only an F*_max_* of 39.4 ± 5.5 N is required, which indicates that, also at these fast conditions, the microgel with the smallest particle size is injectable. The good injectability of the powdered microgel is highly beneficial compared to a bulk GelMA hydrogel, which is impossible to be injected following cross-linking [17].

#### 2.5.2. Flow Sweep

**Figure 11:**
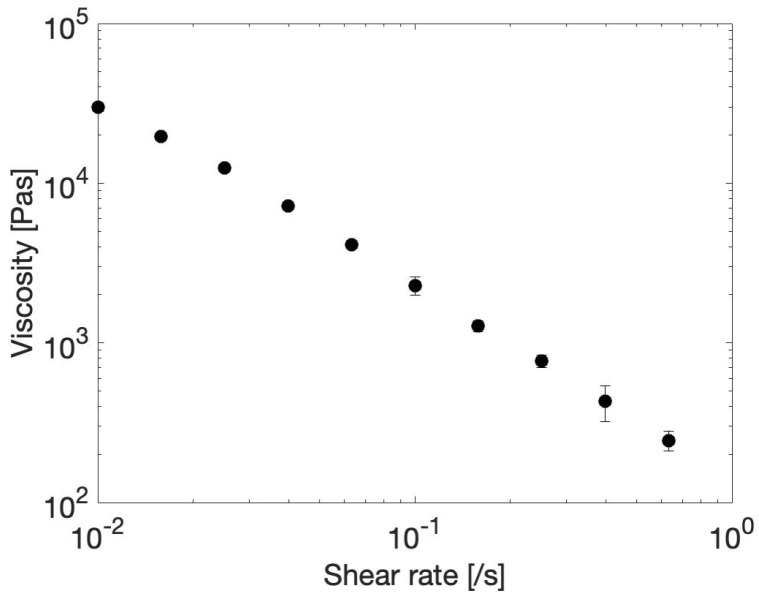
Flow sweep of the 100–212 µm microgel in water at 20 °C, indicating shear thinning behavior. Mean ± standard deviation of two measurements is shown.

More information about the injectability of the 100–212 µm microgel in water is obtained by performing a flow sweep. The flow sweep shows the viscosity of the microgel as a function of shear rate, as shown in Figure 11. It is clear that the microgel exhibits shear thinning behavior, i.e., the viscosity decreases as a function of increasing shear rate. This is advantageous for injection because it means that the resistance to flow decreases as the microgel is pushed through the needle. It can be noted that the slope of viscosity versus shear rate is close to −1 on a double logarithmic scale, which indicates that flow occurs at a nearly constant shear stress of 300 Pa, which is the yield stress of the material.

### 2.6. Cytocompatibility Assays

**Figure 12:**
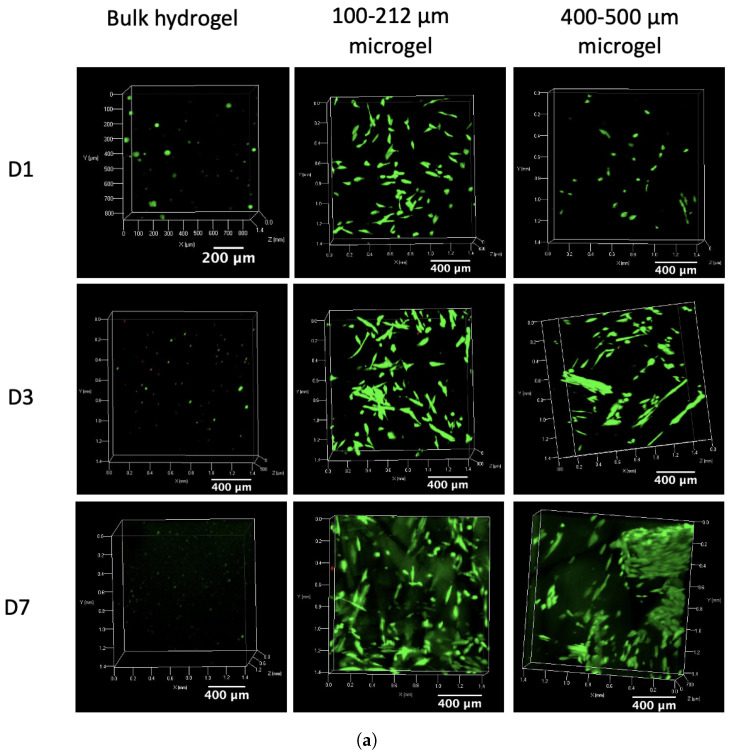

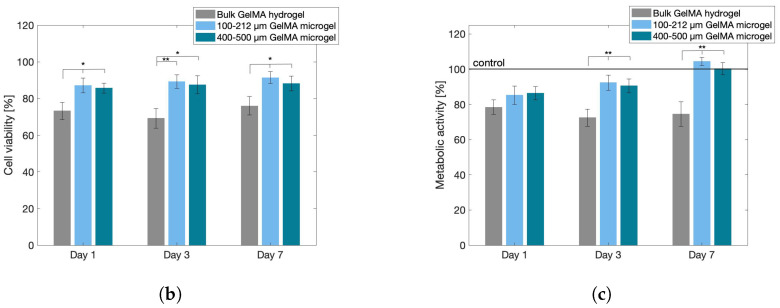
Cytocompatibility of the 100–212 µm and 400–500 µm GelMA microgels compared to that of a bulk GelMA hydrogel evaluated at multiple time points (days 1, 3 and 7). (**a**) Z-stacked confocal images showing both live (green) and dead (red) cells. (**b**) Cell viability according to the live/dead assay using calcein-AM and PI shown as the percentage of live cells to total cells. (**c**) Metabolic activity using an MTS assay relative to the tissue culture plastic control. Mean ± standard deviation of triplicate measurements are presented. Statistically significant differences are indicated by an asterisk (* = *p* < 0.05, ** = *p* < 0.01).

The cell viability of ASCs encapsulated in the GelMA microgel suspension was assessed and compared to that of cells encapsulated in a regular bulk GelMA hydrogel of a similar concentration (10 *w*/*v*%) based on live/dead staining with calcein-AM and propidium iodide at different time points (1 day, 3 days, 7 days). The images in Figure 12a show a clear difference in cell viability and morphology of the cells in the microgels compared to those in a bulk hydrogel. The cells in the microgels proliferate over time, eventually also showing spreading towards an elongated shape in line with healthy mesenchymal stem cell morphology [70]. The cells take up the space within the pores between the microgel particles. This is clearly visualized in the image of the 400–500 µm microgel at day 3 in Figure 12a, in which large, black, square particles can be seen in between bright green fluorescent channels. The extension of these cells within the pore channels promotes cell–cell interaction, which enhances cellular growth. Similar results were shown in the work of Seymour et al. [35], who studied the infiltration of HUVECs in a 3D-printed GelMA microgel. They concluded that the large pore sizes in between the microgel particles allowed excellent cell infiltration. The quantification of cell viability, shown in Figure 12b, indicates that more than 88% of the cells were viable after 7 days of encapsulation in both the 100–212 µm and 400–500 µm microgels. Furthermore, the microgel particle size does not affect the cell viability, as validated by the similar cell viability values of both microgels at each of the measured time points. Lastly, a higher metabolic activity could be observed in Figure 12c for the microgel systems compared to the control at day 7, confirming the cytocompatibility of the gel. This higher metabolic activity can be attributed to more cells being present in the 3D gel structure compared to the 2D tissue culture plastic control sample, since the former exposes a larger surface area to grow and thus less contact inhibition will occur, which normally ceases proliferation [71]. In contrast to the microgels, the bulk hydrogel shows limited cell viability, as visualized in Figure 12a and quantified in Figure 12b, and no extension of the cells, resulting in more rounded cells. In addition, the metabolic activity of the cells also reduces over time to a level lower than that of the control. The reduced cell viability is already visible at day 1 in Figure 12c, and the difference compared to the viability of the cells in the microgels remains similar throughout the entire measurement time frame. Therefore, the reduction is expected to occur due to the exposure of the cells to UV-A and the smaller mesh sizes inside the bulk hydrogel compared to the pores in between microgel particles. Indeed, it has been reported in the literature that UV-A irradiation in the presence of cells can lead to chromosomal as well as genetic instability [72,73]. Moreover, reactive oxygen species (ROS) created by the PI cleavage as well as the reaction of UV-A with oxygen can cause oxidative damage to DNA [74,75]. To conclude, the cytocompatibility assays reveal that the microgels are superior compared to the bulk hydrogel for the encapsulation of cells.

## 3. Conclusions

The current work evaluated the potential of a powdered cross-linked GelMA microgel as an injectable scaffold material for tissue engineering applications. For this, the rheological properties at physiological conditions, injectability and cytocompatibility of the gel were extensively examined. The chemical modification of gelatin into GelMA was proven to be successful, with an obtained DS of 63% and only a minor molar mass reduction of approximately 13%. However, the modification did complicate the formation of hydrogen bonds necessary to stabilize triple helices. As a consequence, only 50% of the physical cross-links present in the non-modified gelatin were able to form in the modified gel. The chemical cross-links ensured that the material could maintain its gel character at temperatures above the sol–gel transition of pristine gelatin. Since only a limited number of physical cross-links were present, the stiffness reduction related to the unfolding of the triple helices at temperatures above 30 °C was minimal. On the contrary, the presence of salts caused a pronounced reduction in the stiffness of the particulate hydrogel. This occurred due to the deswelling of the particles, which was presumably driven by the shielding of short-range electrostatic interactions, leading to the aggregation of the polymer chains and the expulsion of water out of the particles as a consequence of salting out. Moreover, at high salt contents, more easily deformable particles were generated by the denaturation of their triple helices. Furthermore, it was proven that the rheological properties were similar for gels consisting of different particle sizes. To verify the capability of translation for clinical use, the injectability of the gel through a 20G needle was examined. The force necessary to induce a steady flow of the microgel constituting particles smaller than 212 µm out of the syringe was found to be easily achieved by human manipulation. Finally, the cytocompatibility of the microgel was proven to be superior to that of a bulk hydrogel by performing live/dead staining with calcein-AM and propidium iodide and through a metabolic activity assay of encapsulated ASCs. In addition, particle size did not affect the viability or the metabolic activity of the cells. Moreover, the powdered GelMA microgel is not only superior to the bulk GelMA hydrogel in terms of cytocompatibility but also based on its mechanical properties. An elastic modulus similar to that of actual adipose tissue is achieved for the microgel. Furthermore, the mechanical properties of the microgel are more easily adapted than those of the bulk gel due to the ability to change the volume fraction of the particles. Even though the shape of the powder particles is complex, the production procedure allows a high throughput, which is a significant advantage compared to other microgel production procedures. In conclusion, it can be stated that powdered cross-linked gelatin methacryloyl is a highly promising candidate for use as a cost-effective scaffold material in adipose tissue engineering, having superior properties compared to a bulk GelMA hydrogel.

## 4. Materials and Methods

### 4.1. Materials

Gelatin type B (GelB), isolated from bovine skin, was kindly supplied by Rousselot (Ghent, Belgium). Methacrylic anhydride, bovine serum albumin (BSA), cryopreserved adipose tissue-derived stem cells (human adipose mesenchymal stem cells, SCC038), calcein-acetoxymethyl (calcein-AM), propidium iodide (PI) and butyldiglycol acetate were purchased from Merck (Diegem, Belgium). An MTS assay kit was purchased from Abcam (Cambridge, UK). The Spectrapor dialysis membranes (MWCO 12,000–14,000 Da) were purchased from Polylab (Antwerp, Belgium). Dulbecco’s modified Eagle’s medium (DMEM), fetal bovine serum (FBS), 1% penicillin/streptomycin and Trypsin were obtained from Gibco (Life Technologies, Carlsbad, CA, USA).

Solutions of sodium chloride (Acros Organics, Geel, Belgium) in double-distilled water (ddH_2_O, σ < 4 µS/cm) were prepared at 1 and 10 mM. A phosphate buffered saline (PBS, 0.01 M phosphate buffer, 0.0027 M potassium chloride and 0.137 M sodium chloride, pH 7.4, at 25 °C) tablet (Merck, Diegem, Belgium) was dissolved in 200 mL ddH_2_O, and the solution was diluted to 67.5% of its initial concentration. The latter was needed to remain below the upper conductivity limit of the electrokinetic analyzer. The ionic strengths, conductivities, osmolalities and pH values of these three salt solutions are shown in Table 3, in addition to those of water. These salt solutions were chosen based on their simplicity and their ability to mimic the fluid of the ECM. Sodium is one of the main constituents of the ECM, and PBS is often used in physiological studies [52]. The current study focuses on the effect of the ionic strength on the rheological properties of the microgel particles. Therefore, the ionic strength of the solutions varied from zero for pure water to 110 mM, which is close to the actual physiological ionic strength of approximately 155 mM [76]. To avoid cell damage due to high osmolality, the osmolality values of the solvents were checked. These values are well below the osmolality of lipoaspirate, which is stated to be 315 mOsm/L by Potočar et al. [77], such that no cell damage by the solvents is expected.

### 4.2. Synthesis of Gelatin Methacryloyl

The methacrylation of gelatin type B (resulting in GelMA) was performed as described in previous reports of Van Den Bulcke et al. [14]. In brief, 100 g of GelB (38.5 mmol primary amines) was dissolved in phosphate buffer (1 L, pH 7.8) at 40 °C under continuous mechanical stirring. Next, 1 equivalent of methacrylic anhydride (relative to the amount of primary amines) was added and left to react for 1 h. Following reaction, 1 L of ddH_2_O was added, and the solution was subsequently dialyzed (MWCO 12,000–14,000 Da) for 24 h at 40 °C. To this end, the water was changed five times, followed by lyophilization of the obtained solution (Christ freeze-dryer alpha I-5). The degree of substitution (DS) of GelMA was quantified via ^1^H-NMR spectroscopy (Bruker WH 500 MHz) using deuterium oxide (D_2_O, 10 mg/mL) as the solvent at 40 °C.

### 4.3. Characterization of GelMA Precursors

#### 4.3.1. Gel Permeation Chromatography (GPC)

GPC measurements were performed on a Waters 610 fluid unit and a Waters 600 control unit equipped with a Waters 410 RI detector and SB-806M HQ column (Shodex). Samples were prepared by dissolving approximately 10 mg of material in a buffer (pH 7) containing 0.01 M NaH_2_PO_4_ and 0.2 M NaNO_3_. A four-point calibration curve was prepared using pullulan standards (M_peak_ = 47,100; 107,000; 337,000; 642,000 Da).

#### 4.3.2. Differential Scanning Calorimetry (DSC)

GelB and GelMA solutions (10 *w*/*v*%, 40 mg, ddH_2_O) were placed in a hermetic Tzero pan (TA Instruments, Zellik, Belgium). An empty hermetic Tzero pan was used as reference. All measurements were performed on a TA Instruments Q 2000 with an RSC 500 cooler. The samples were subjected to a preparatory program, as described by Prado and Vyazovkin [78], who used this protocol to measure the thermal properties of gelatin-like materials. To this end, a temperature ramp of 20 °C/min was applied until a temperature of 60 °C was reached. The sample was then stabilized for 20 min. Subsequently, the sample was cooled to 15 °C at a rate of 10 °C/min, followed by a stabilization phase for 20 min. Next, a temperature ramp of 20 °C/min was applied until a temperature of −10 °C was reached. Lastly, the samples were heated again at a rate of 5 °C/min until a temperature of 60 °C was reached.

### 4.4. Processing into Swellable Powder

First, a 10 *w*/*v*% solution of the above synthesized material was dissolved at 40 °C in a vial with ddH_2_O. Following complete dissolution, 0.6 mmol of a photo-initiator (lithium phenyl-2,4,6-trimethylbenzoylphosphinate; LAP) was added. The material was then placed between two glass plates, separated with a 1 mm spacer and put under UV-A (±9.5 mW/cm^2^) light for 30 min. The total UV-A dose thus amounted to 17.1 J/cm^2^. The resulting UV dose was verified to be more than double the required amount for full cross-linking, thereby avoiding batch-to-batch variability. Following UV curing, the samples were frozen, followed by freeze-drying and grinding. The grinding was performed manually using a mortar. In the grinding step, liquid nitrogen was used to increase the brittleness of the material. More details on the exact production procedure for the powder particles can be found in the patent WO2021255295 [79]. Next, the powder particles were manually sieved to obtain the following five size fractions, with the sizes indicating the mesh size of the sieves in between which the particles were retained: <100 µm, 100–212 µm, 212–300 µm, 300–400 µm and 400–500 µm. It should be noted that the particles have a rectangular shape, as shown in the Supplementary Information (Figures S2–S6), such that these size fractions are based on the length of the smallest side of the rectangular particle. To finally obtain particulate gels, powder particles of a certain size fraction were dissolved in a solvent of interest at 10 *w*/*v*%.

### 4.5. Rheological Characterization

Rheological measurements were carried out on a stress-controlled MCR702 rheometer (Anton Paar GmbH, Graz, Austria) equipped with a home-made roughened aluminum plate screwed onto a Peltier plate (Anton Paar GmbH, Graz, Austria) and a 25 mm diameter roughened aluminum top plate geometry. The sample was surrounded by a thin layer of mineral oil to avoid dehydration. To rule out effects of confinement and particle compression, each hydrogel sample was measured at a gap height of at least three times the maximum size of a dry powder particle of that size fraction. This gap height was well above the critical confinement gap of microgel particles, which is defined by Vleminckx et al. [80] to be 2D, with D the diameter of the particle. The hydrogel samples were prepared at a concentration of 10 *w*/*v*% in water and in the three salt solutions (see Table 3), and left to rest 2 h prior to the measurement, such that the particles could reach their equilibrium swelling. First, a time sweep of one hour was performed at a strain of 0.1% and a frequency of 10 rad/s to ensure that the sample had achieved steady-state conditions. Next, a frequency sweep at a constant strain of 0.1% was carried out from 100 to 0.1 rad/s. The strain of 0.1% was chosen to be inside the linear viscoelastic (LVE) region, as determined from a strain sweep performed at 10 rad/s. All these measurements were performed isothermally at a temperature of 20 °C. Measurements were performed twice for the 100–212 µm and 212–300 µm size ranges to define reference standard deviations, which are indicated as error bars on the figures.

To investigate the viscoelastic stability of the microgels at body temperature, a time sweep of 15 h (at 0.1% strain and 10 rad/s) followed by a frequency sweep (at 0.1% strain) was performed on a microgel made from the 212–300 µm particle size fraction at 37 °C. In addition, a temperature sweep at 0.1% strain and 10 rad/s was performed for each particle size fraction to probe the temperature-dependent rheological properties between 18 and 45 °C at a heating and subsequent cooling rate of 0.85 °C/min.

A flow sweep from 0.01 to 10/s was performed to assess the shear thinning behavior of the 100–212 µm gel in water. Each point was taken when a steady state was reached. It should be noted that only the data in the 0.01 to 1/s range are shown, since edge fracture caused the material to be pushed out from underneath the geometry at shear rates higher than 1/s.

### 4.6. Structural Characterization

#### 4.6.1. Laser Diffraction

The swelling of particles in water and in multiple salt solutions was quantified by means of an LS 13 320 particle size analyzer (Beckmann Coulter, Miami, FL, USA) equipped with a 750 nm laser. Particles were suspended in water, 1 mM NaCl, 10 mM NaCl, diluted PBS or butyldiglycol acetate at a concentration of 1 mg/mL, and left to rest on a rolling plate for 2 h until the measurement. The latter solvent was used to create an unswollen particle suspension since its hydrophobic character does not allow it to penetrate inside the hydrophilic particles. It was confirmed via brightfield microscopy that the particles did not swell in butyldiglycol acetate. The liquid module of the particle size analyzer was filled with the solvent of interest, to which 10 mL of the respective particle suspension was added. The scattering intensity was recorded and translated into particle size distributions according to the Fraunhofer theory using the instrument’s software. The volumetric swell factor was calculated according to

(1) $$SF=A_{s}/A_{\mathrm{us}}$$

with $A_{s}$ and $A_{\mathrm{us}}$ the areas of the volumetric particle size distribution curves of particles swollen in water or in a salt solution and unswollen in butyldiglycol acetate, respectively. Results are presented as mean ± standard deviation of four measurement runs for each solvent.

#### 4.6.2. Micromechanics

A micromechanics setup based on the design of Guo and Wyss [81] was used to determine the compression modulus of single gel particles. The main part of this setup consists of a tapered glass capillary, which was obtained by pulling a glass capillary with a 1.2 mm outer diameter and a 0.9 mm inner diameter with a micropipette puller (Model P-97, Sutter Instrument, Novato, CA, USA) to obtain a tip opening of 60 µm. The capillary was coated with 2 wt% BSA to eliminate frictional resistance between the particles and the glass wall [82]. The inlet of the capillary was initially connected to a syringe containing 212–300 µm particles suspended in a solvent of choice (water or salt solutions, see Table 3) via flexible tubing. By manual manipulation of the syringe plunger, the particles were flushed towards the tip of the capillary until one particle was captured inside the tip, thereby completely blocking the flow. The tip of the capillary was constantly submerged inside a fluid bath containing the same solvent as the particle suspension to maintain a stable hydrodynamic pressure. Once the particle was stabilized inside the tip, the tubing was disconnected from the syringe and connected to air pressurized at 0.5 bar. Next, the pressure was stepwise increased up to 4.5 bar in steps of 0.5 bar, allowing the particle to deform and reach its equilibrium shape at each of these pressures. For each pressure, the particle’s image was captured by means of an Olympus BX51WI upright light microscope (Olympus, Tokyo, Japan) connected to a Hamamatsu C4742–95 camera (Hamamatsu Photonics K. K., Hamamatsu City, Japan). From these images, the geometrical parameters indicated in Figure 8b were obtained by manually measuring them in ImageJ (1.53i). Next, the compression modulus K was calculated as the slope of (2$p_{\mathrm{wall}}$ + *p*)/3 plotted against ΔV/V. The average wall pressure, $p_{\mathrm{wall}}$ and the volumetric strain deformation, ΔV/V, are defined according to Equations (2) and (3), respectively [82]:

(2) $$p_{\mathrm{wall}}=\frac{2}{sin(\alpha )}\frac{R_{\mathrm{band}}}{L_{\mathrm{band}}}p$$

(3) $$\Delta V/V=\frac{\pi r_{0.5\mathrm{bar}}^{2}h_{0.5\mathrm{bar}}-\pi r_{p}^{2}h_{p}}{\pi r_{0.5\mathrm{bar}}^{2}h_{0.5\mathrm{bar}}}=\frac{r_{0.5\mathrm{bar}}A_{0.5\mathrm{bar}}-r_{p}A_{p}}{r_{0.5\mathrm{bar}}A_{0.5\mathrm{bar}}}$$

with α, $R_{\mathrm{band}}$, $L_{\mathrm{band}}$, $r_{\mathrm{front}}$ and $r_{\mathrm{back}}$ indicated in Figure 8b. $A_{0.5\mathrm{bar}/p}$ and $h_{0.5\mathrm{bar}/p}$ are the area and height (longest side of the rectangle) of the swollen particle at 0.5 bar or at the subsequently applied pressures. Equation 3 is the result of approximating the particle’s shape with a cylinder. The radius of the cylinder at 0.5 bar, $r_{0.5\;\mathrm{bar}}$, or at one of the subsequent pressures, $r_{p}$, is taken as the average of the front radius, $r_{\mathrm{front}}$, and back radius, $r_{\mathrm{back}}$, of the particle. Results are presented as mean ± standard deviation of at least five particles for each solvent.

#### 4.6.3. Circular Dichroism (CD)

A Jasco J-810 spectropolarimeter (Jasco, Tokyo, Japan) was used to obtain the circular dichroism (CD) spectra of 100–212 µm particles in water and in the three salt solutions (see Table 3). The particle size range of 100–212 µm was chosen to minimize the sedimentation of the particles inside the cuvette. The suspensions were prepared at a concentration of 0.8 mg/mL 2 h prior to the measurements to allow complete swelling of the particles. A 1 cm path length quartz cuvette was filled with the suspension, and spectra were taken from 260 to 180 nm at a scanning speed of 200 nm/min and a bandwidth of 1 nm. Each sample was measured three times to check reproducibility. This proved to be excellent (see Figure S13 in the Supplementary Information), so only the first scan is displayed for each solvent. The results are presented with solvent background subtraction and binomial smoothing. CD values corresponding to a high-tension voltage higher than 700 V are not incorporated in the presented spectra [83].

#### 4.6.4. Zeta Potential

An electrokinetic analyzer (SurPASS 3, Anton Paar GmbH, Graz, Austria) was used to measure the zeta potential of 100–212 µm powder particles in water and in the three different salt solutions (see Table 3). The powder, which was obtained as a granular sample, was loaded in a measuring cell suitable for powder samples > 25 µm (Cylindrical cell, Anton Paar GmbH, Graz, Austria). Prior to the measurements, the solvent was circulated through the device to adjust the permeability index to 100 and, at the same time, allow the particles to swell to their equilibrium size. Next, the streaming potential was recorded during five measurement cycles under specific pressure settings (upper pressure: 600 mbar, lower pressure: 200 mbar, cut-off pressure: 100 mbar), and the zeta potential for each cycle was calculated using the Helmholtz–Smoluchowski equation. The obtained zeta potential values of the five measurement cycles on a single sample per solvent are reported as mean ± standard deviation.

### 4.7. Injectability: Force–Displacement Measurement

The injectability of the material was tested using a universal testing machine (Tinius Olsen, Redhill, UK) equipped with a 500 N load cell. To this end, the powder particles swollen in water were loaded in a 1 mL syringe (1.9 mm tip diameter), optionally equipped with a 20G needle (inner diameter 603 µm), and placed in a syringe holder. A preload of 1 N and speed of 5 or 10 mm/min were used to measure the maximal load, load at plateau and stiffness. The measurements were performed in duplicate, and mean ± standard deviation is reported.

### 4.8. Cytocompatibility

The cytocompatibility of the gel was assessed at different time points (days 1, 3 and 7) through both a live/dead assay using calcein-acetoxymethyl (calcein-AM) and propidium iodide (PI) and an MTS assay. First, the powder particles were sterilized by placing them under UV-C for 1 h. Next, sterilized PBS was added to the particles to obtain microgels at 10 *w*/*v*% and each microgel was transferred to a 96-well plate at 100 µL per well. Subsequently, adipose tissue-derived mesenchymal stem cells (ASCs) (passage 5) were encapsulated in the microgel by pipetting the cell mixture onto the gel particles at a density of 1,000,000 cells per mL and gently mixing with a spatula. Likewise, a bulk GelMA precursor solution was prepared by dissolving GelMA in sterilized PBS at a concentration of 10 *w*/*v*% in a water bath at 37 °C. ASCs at a density of 1,000,000 cells per mL were added to the precursor solution, and the solution was transferred to a 96-well plate at 100 µL per well. Next, the plate was placed in the fridge for 15 min to allow physical gelation, after which it was exposed to UV-A for 15 min to chemically cross-link the cell-containing GelMA gels. For each gel (microgel and bulk hydrogel), six wells were prepared to perform triplicate measurements of the live/dead assay as well as the MTS assay. In addition, a control was prepared by pipetting 100 µL of the cells at a density of 1,000,000 cells per mL in the 96-well plate. Lastly, 200 µL of basic culture medium, consisting of high glucose DMEM, 1% penicillin/streptomycine and 10% FBS, was added to the well plates.

#### 4.8.1. Live/Dead Assay

The cytocompatibility of the materials was tested through a live/dead viability assay using calcein acetoxymethyl ester (calcein-AM) and propidium iodide (PI) staining. For every 1 mL PBS, 2 µL calcein-AM and 2 µL PI were added. An aliquot of 150 µL of this solution was added to three wells of the previously prepared 96-well plates for each hydrogel. The wells were incubated in the dark by placing them under aluminum foil for 20 min at room temperature. A confocal microscope with a green fluorescent protein (GFP) filter for calcein was used to visualize the living cells. A Texas Red (TxRed) filter was applied to visualize the dead cells using PI. The confocal images were retrieved in a z-stack and are therefore also shown as stacked images. The quantification of the live/dead ratio was realized via ImageJ software (1.53i), enabling a cell count of both living and dead cells. The percentage of live cells to total cells is presented as mean ± standard deviation of triplicate measurements.

#### 4.8.2. MTS Assay

An 3-(4,5-dimethylthiazol-2-yl)-5-(3-carboxymethoxy phenyl)-2-(4-sulfophenyl)-2H-tetrazolium (MTS) assay kit was used to assess the cell proliferation. The basic culture medium of the cells in the remaining wells (those that were not used for live/dead staining) was discarded and refreshed with 100 µL medium. The well plates were topped with 20 µL of MTS solution and incubated in the dark (wrapped in aluminum foil) on a shaker at 37 °C for 1.5 h. Next, 100 µL of the supernatants was added to a fresh 96-well plate and placed on a plate reader measuring the absorbance (at 580 nm) using a spectrophotometer (Bio-Tek EL800). The percentage of metabolic activity of the cells inside the gels relative to that of the cells in the control is presented as mean ± standard deviation of triplicate measurements.

### 4.9. Statistics

For replicate samples, outlier values were calculated from the residuals versus group means plot using a one-way analysis of variance in R and were not incorporated in the resulting bar graphs. An unpaired *t*-test was performed to determine if two average values were significantly different, considering a *p*-value smaller than 0.05 (* = *p* < 0.05, ** = *p* < 0.01, *** = *p* < 0.001, **** = *p* < 0.0001).

## 5. Patents

The authors Sandra Van Vlierberghe and Lana Van Damme are the inventors of the pending patent #WO2021255295A1: Swellable gelatin compositions.

## Figures and Tables

**Table 1:** Summary of the most commonly applied microgel fabrication techniques and their main drawbacks.

Technique	Drawback	References
Microfluidics	Need for an oil phase, which might impart biocompatibility	[30]
	Low throughput	[30]
	Need for deep cleaning of the device	[36]
Emulsification	Need for an oil phase, which might impart biocompatibility	[30]
	High polydispersity	[30]
Electrostatic droplet generation	High polydispersity	[36]
Two-step desolvation	Use of toxic solvents	[37]
Complex coacervation	Agglomeration and stabilization issues	[38]
Lithography	Low throughput	[30]
	Physical removal of molds leading to deformations	[36]
Mechanical fragmentation	High polydispersity	[30]
(in the wet state)		

**Table 2:** Aspect ratio defined as the ratio of the height (*H*) and the width (*W*) of the water-swollen GelMA powder particles. Mean ± standard deviation of at least four particles are presented.

Size Fraction	Aspect Ratio $\frac{H}{W}$ (-)
<100 µm	4.51 ± 1.81
100–212 µm	3.79 ± 0.69
212–300 µm	1.73 ± 0.24
300–400 µm	1.46 ± 0.31
400–500 µm	1.36 ± 0.24

**Table 3:** Ionic strength, conductivity, osmolality and pH of the applied solvents: water, 1 mM NaCl, 10 mM NaCl and diluted PBS.

Solvent	Ionic Strength (mM)	Conductivity (mS/cm)	Osmolality (mOsm/L)	pH
Water	≈0	≈0	0	6.39
1 mM NaCl	1	0.36	2	7.07
10 mM NaCl	10	1.52	20	7.25
Diluted PBS	110	8.85	243	7.57

**Table 4:** Volume fractions of particles in the 212–300 µm size range swollen in 10 *w*/*v*% gels based on water, 1 mM NaCl, 10 mM NaCl and diluted PBS, respectively.

Solvent	Volume Fraction (%)
Water	104
1 mM NaCl	59.8
10 mM NaCl	48.0
Diluted PBS	31.4

## Data Availability

The data presented in this study are openly available in article.

## Supplementary Materials

The following supporting information can be downloaded at: https://www.mdpi.com/article/10.3390/gels10030167/s1, Figure S1: ^1^H-NMR spectrum of the methacryloyl modified gelatin (GelMA). The peaks corresponding with the protons of the methacryloyl functionalities are indicated with a. The peak that is marked with b corresponds to the hydrogens from the chemically inert valine, leucine and isoleucine amino acids; Figure S2: Microscopic images showing the rectangular shape of the < 100 µm particles (a–c) in a 10 *w*/*v*% gel and (d,e) diluted in water; Figure S3: Microscopic images showing the rectangular shape of the 100–212 µm particles (a,b) in a 10 *w*/*v*% gel prior to injection, (c,d) in a 10 *w*/*v*% gel after injection through a 18G needle (inner diameter 838 µm) and (e,f) diluted in water; Figure S4: Microscopic images showing the rectangular shape of the 212–300 µm particles (a,b) in a 10 *w*/*v*% gel and (c,d) diluted in water; Figure S5: Microscopic images showing the rectangular shape of the 300–400 µm particles (a,b) in a 10 *w*/*v*% gel and (c,d) diluted in water; Figure S6: Microscopic images showing the rectangular shape of the 400-500 µm particles (a-b) in a 10 *w*/*v*% gel and (b,c) diluted in water. (e) Image of the swollen gel particles in a 10 *w*/*v*% gel; Figure S7: PSD’s of particles of size fractions < 100 µm, 100–212 µm, 212–300 µm, 300–400 µm and 400–500 µm swollen in water; Figure S8: Raw data of (2$p_{\mathrm{wall}}$ + *p*)/3 plotted against ΔV/V used for the calculation of the compression modulus of particles swollen in (a) water, (b) 1 mM NaCl, (c) 10 mM NaCl and (d) diluted PBS; Figure S9: Raw data obtained during zeta potential measurements indicating the unstable measurement of particles swollen in water compared to salt solutions; Figure S10: Time sweep at 1% strain and 10 rad/s of a bulk GelMA hydrogel of 10 *w*/*v*% in water at room temperature; Figure S11: Strain sweep at 10 rad/s of a microgel constituting of 212–300 µm particles in water at 20 °C and 37 °C indicating that temperature has no effect on the yielding; Figure S12: Force versus displacement curves of 10 *w*/*v*% hydrogels in water constituting of the 212–300 µm, 300–400 µm and 400–500 µm particle size fractions that were pushed through a syringe of 1.9 mm tip diameter in 42 s; Figure S13: Triplicate spectra showing good reproducibility of 100–212 µm particles swollen in diluted PBS; Video S1: Injectability.

## Abbreviations

The following abbreviations are used in this manuscript:

ASCs	Adipose tissue-derived stem cells
BSA	Bovine serum albumin
calcein-AM	calcein-acetoxymethyl
CD	Circular dichroism
DMEM	Dulbecco’s modified Eagle’s medium
DNA	Deoxyribonucleic acid
DS	Degree of substitution
DSC	Differential scanning calorimetry
ECM	Extracellular matrix
FBS	Fetal bovine serum
GelB	Gelatin type B
GelMA	Gelatin methacryloyl
GFP	Green fluorescent protein
GPC	Gel permeation chromatography
HUVECs	Human umbilical vein endothelial cells
LAP	Lithium phenyl-2,4,6-trimethylbenzoylphosphinate
LVE	Linear viscoelastic
MA	Methacryloyl
MM	Molar mass
MTS	3-(4,5-dimethylthiazol-2-yl)-5-(3-carboxymethoxyphenyl)-
	2-(4-sulfophenyl)-2H-tetrazolium
NMR	Nuclear magnetic spectroscopy
PBS	Phosphate buffered saline
PI	Propidium iodide
PSD	Particle size distribution
RGD	Arg-Gly-Asp amino acid triplet
ROS	Reactive oxygen species
TE	Tissue engineering
TxRed	Texas Red
UCST	Upper critical solution temperature
UV	Ultraviolet
