# Supplementary material for: Powdered Cross-Linked Gelatin Methacryloyl as an Injectable Hydrogel for Adipose Tissue Engineering
Source: Gels. 2024 Feb 26;10(3):167. doi: 10.3390/gels10030167 (PMC10970257; doi:10.3390/gels10030167)
Supplement: Supplementary file 1 [file gels-10-00167-s001.zip › DeMaeseneer-SI/DeMaeseneer-SI.pdf]

Powdered Cross-linked Gelatin Methacryloyl as an Injectable Hydrogel for Adipose Tissue Engineering

Tess De Maeseneer,[a‡] **Lana Van Damme,**[b‡] **Merve Kübra Aktan,**[c] **Annabel Braem,**[c] **Paula Moldenaers,**[a] **Sandra Van Vlierberghe** [b] **and Ruth Cardinaels**[*a,d]

[a] KU Leuven Department of Chemical Engineering, Soft Matter, Rheology and Technology, Celestijnenlaan 200J box 2424, 3001 Leuven, Belgium.
[b] Polymer Chemistry & Biomaterials Group, Centre of Macromolecular Chemistry, Department of Organic and Macromolecular Chemistry, Ghent University (UGent), Krijgslaan 281, S4-Bis, 9000 Ghent, Belgium.
[c] KU Leuven Department of Materials Engineering, Biomaterials and Tissue Engineering research group, Kasteelpark Arenberg 44 box 2450, 3001 Leuven, Belgium.
[d] Processing and Performance of Materials, Department of Mechanical Engineering, TU Eindhoven, P.O. Box 513, 5600 MB Eindhoven, The Netherlands.
* email: ruth.cardinaels@kuleuven.be or r.m.cardinaels@tue.nl

<u>**Supplementary Information**</u>

S1 ^{1}H-NMR spectrum of the methacryloyl modified gelatin (GelMA)

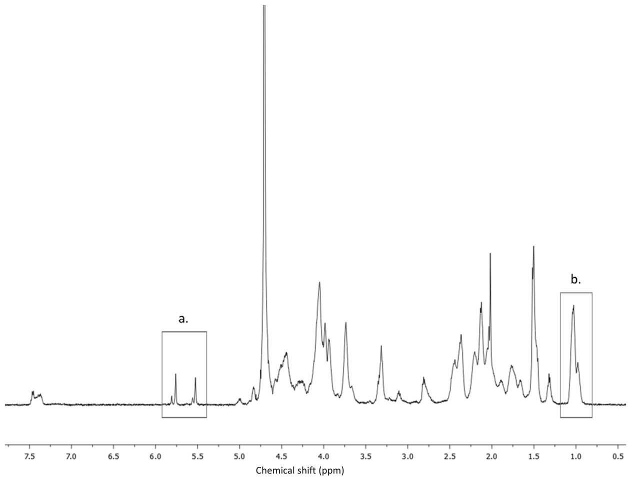

Figure S1: ^{1}H-NMR spectrum of the methacryloyl modified gelatin (GelMA). The peaks corresponding with the protons of the methacryloyl functionalities are indicated with a. The peak that is marked with b corresponds to the hydrogens from the chemically inert valine, leucine and isoleucine amino acids.

S2 Microscopic images of the GelMA powder particles

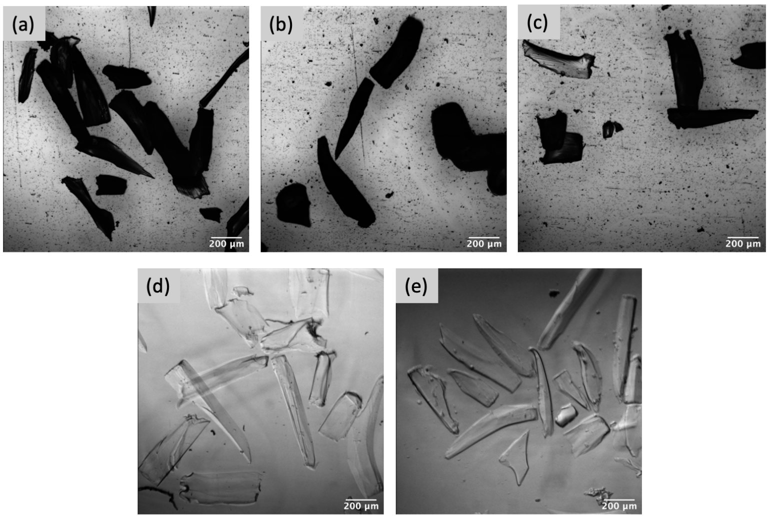

Figure S2: Microscopic images showing the rectangular shape of the < 100 µm particles (a-c) in a 10 w/v% gel and (d-e) diluted in water.

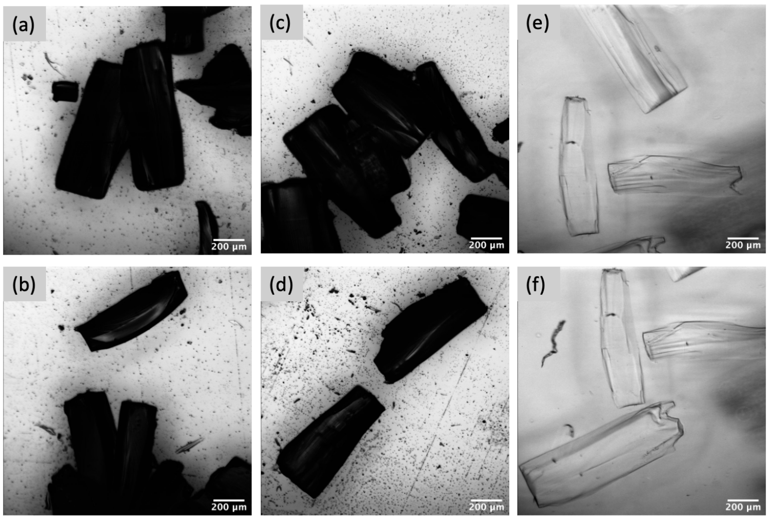

Figure S3: Microscopic images showing the rectangular shape of the 100-212 µm particles (a-b) in a 10 w/v% gel prior to injection, (c-d) in a 10 w/v% gel after injection through a 18G needle (inner diameter 838 µm) and (e-f) diluted in water.

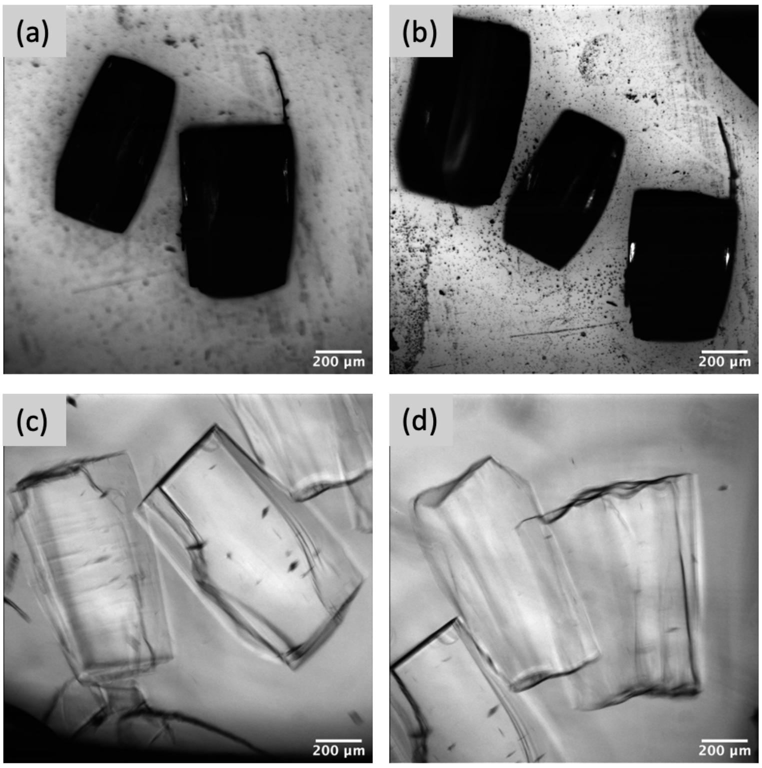

Figure S4: Microscopic images showing the rectangular shape of the 212-300 µm particles (a-b) in a 10 w/v% gel and (c-d) diluted in water.

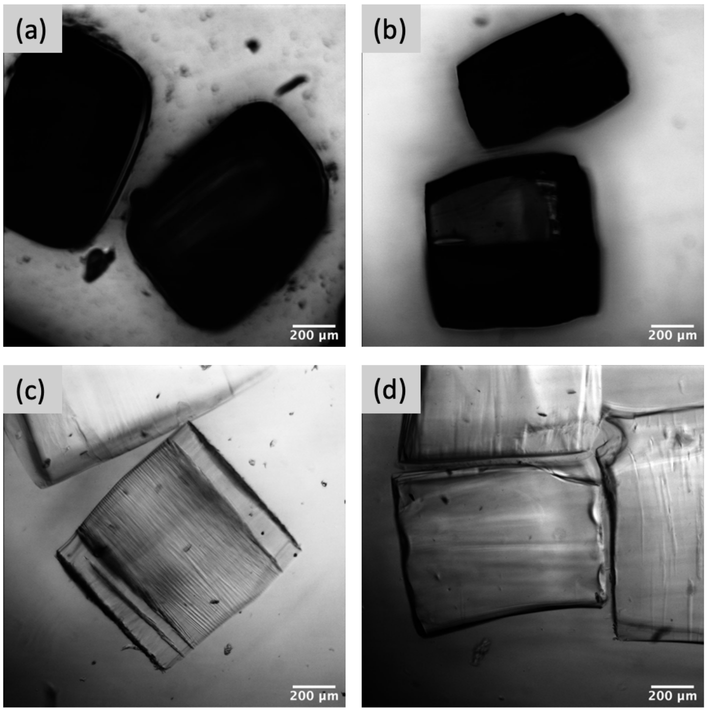

Figure S5: Microscopic images showing the rectangular shape of the 300-400 µm particles (a-b) in a 10 w/v% gel and (c-d) diluted in water.

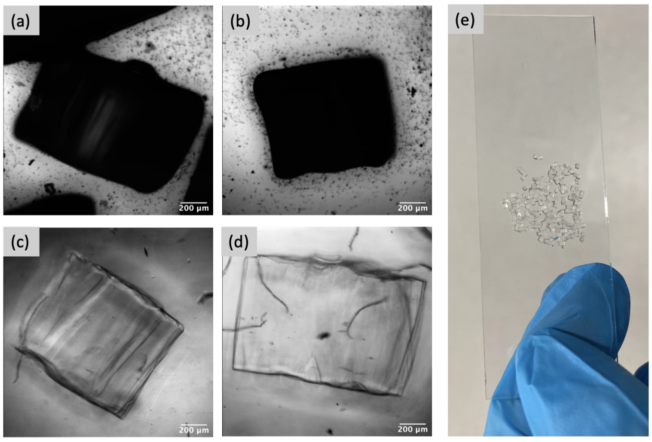

Figure S6: Microscopic images showing the rectangular shape of the 400-500 µm particles (a-b) in a 10 w/v% gel and (b-c) diluted in water. (e) Image of the swollen gel particles in a 10 w/v% gel.

S3 PSD's of the water-swollen GelMA particles

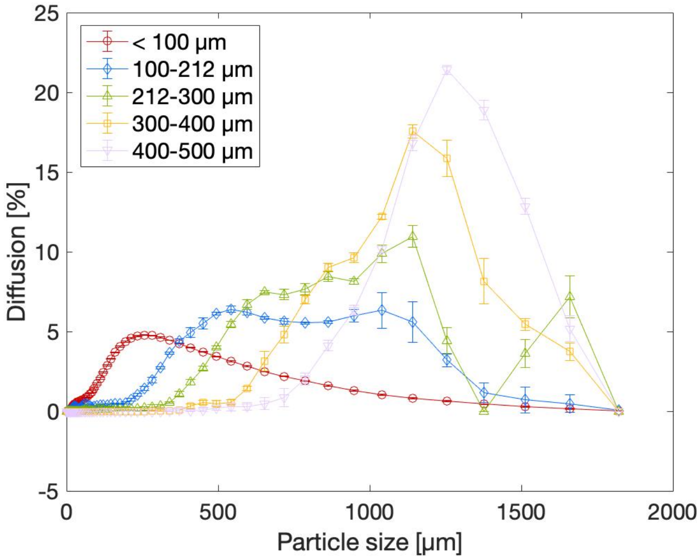

Figure S7: PSD's of particles of size fractions < 100 µm, 100-212 µm, 212-300 µm, 300-400 µm and 400-500 µm swollen in water.

S4 Raw data of the micromechanics experiments

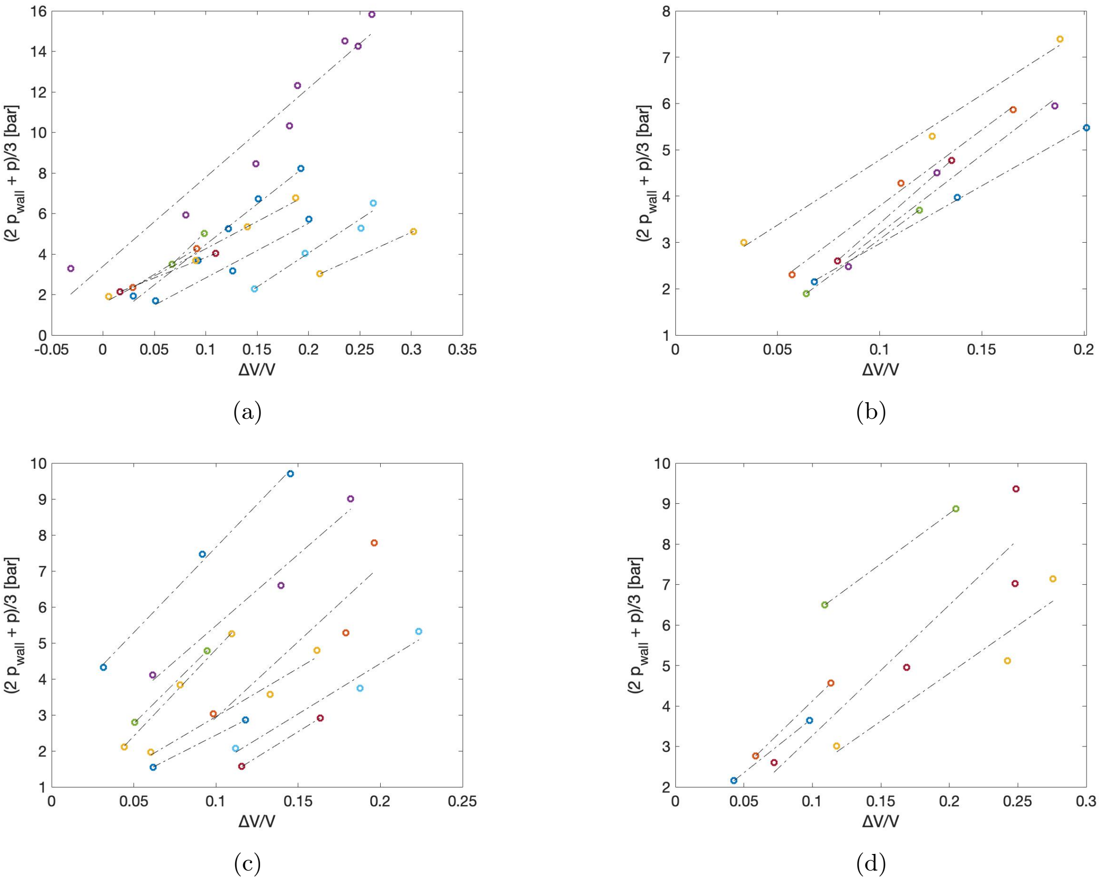

Figure S8: Raw data of $(2p_{\mathrm{wall}} + p)/3$ plotted against $\Delta V/V$ used for the calculation of the compression modulus of particles swollen in (a) water, (b) 1 mM NaCl, (c) 10 mM NaCl and (d) diluted PBS.

S5 Raw data of the zeta potential experiments

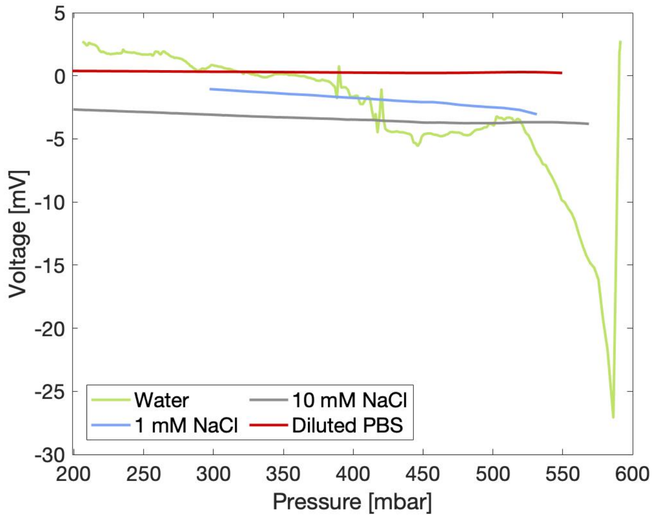

Figure S9: Raw data obtained during zeta potential measurements indicating the unstable measurement of particles swollen in water compared to salt solutions.

S6 Time sweep of a 10 w/v% bulk GelMA hydrogel

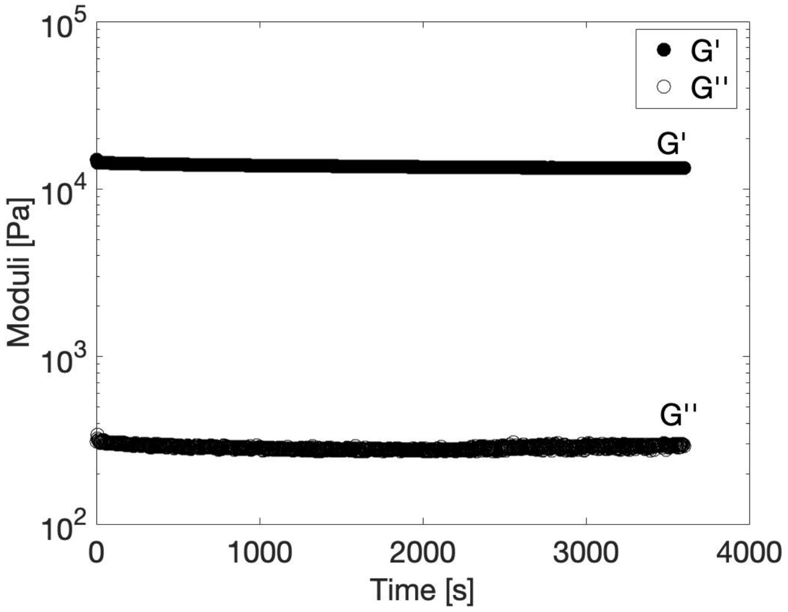

Figure S10: Time sweep at 1 % strain and 10 rad/s of a bulk GelMA hydrogel of 10 w/v% in water at room temperature.

S7 Strain sweep of the 212-300 µm microgel at various temperatures

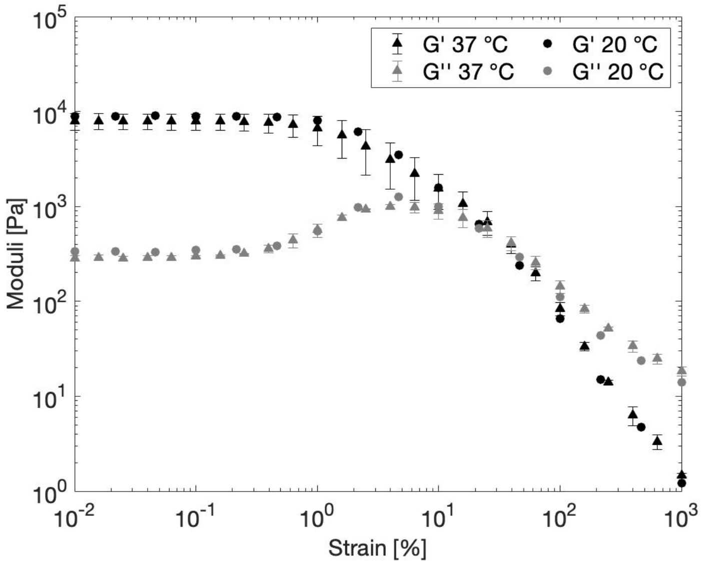

Figure S11: Strain sweep at 10 rad/s of a microgel constituting of 212-300 µm particles in water at 20 °C and 37 °C indicating that temperature has no effect on the yielding.

S8 Injectability of the GelMA microgels

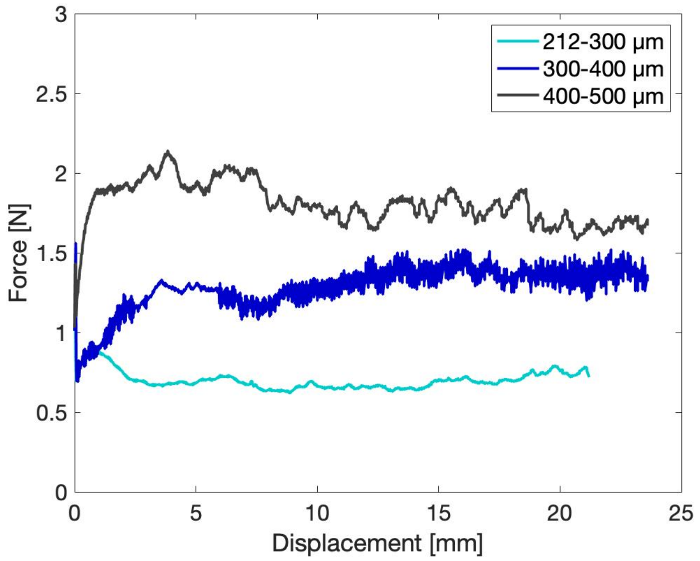

Figure S12: Force versus displacement curves of 10 w/v% hydrogels in water constituting of the 212-300 µm, 300-400 µm and 400-500 µm particle size fractions that were pushed through a syringe of 1.9 mm tip diameter in 42 s.

S9 CD reproducibility of the diluted PBS-swollen GelMA particles

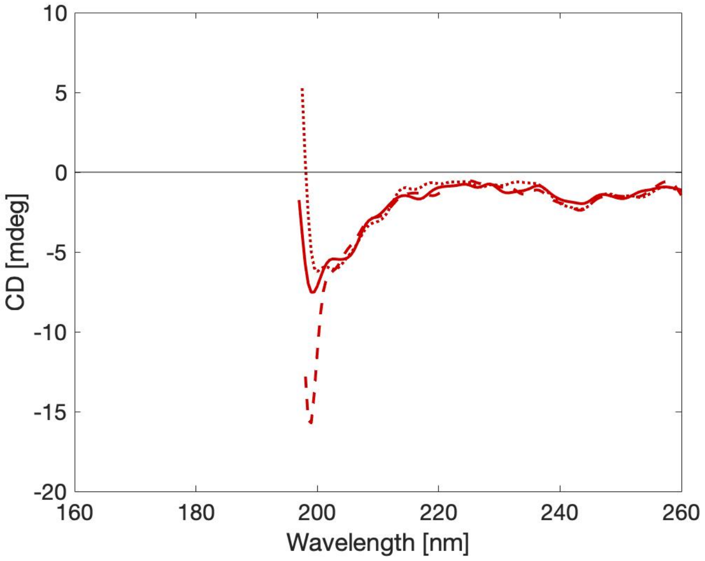

Figure S13: Triplicate spectra showing good reproducibility of 100-212 µm particles swollen in diluted PBS.
